# An image-based, dual fluorescence reporter assay to evaluate the efficacy of shRNA for gene silencing at the single-cell level

**DOI:** 10.12688/f1000research.3-60.v1

**Published:** 2014-02-19

**Authors:** Shin-ichiro Kojima, Gary G. Borisy

**Affiliations:** 1 Department of Life Science, Faculty of Science, Gakushuin University, Tokyo, 171-8588, Japan; 2 The Forsyth Institute, Cambridge, MA, MA 02142, USA

## Abstract

RNA interference (RNAi) is widely used to suppress gene expression in a specific manner. The efficacy of RNAi is mainly dependent on the sequence of small interfering RNA (siRNA) in relation to the target mRNA. Although several algorithms have been developed for the design of siRNA, it is still difficult to choose a really effective siRNA from among multiple candidates. In this article, we report the development of an image-based, quantitative, ratiometric fluorescence reporter assay to evaluate the efficacy of RNAi at the single-cell level. Two fluorescence reporter constructs are used. One expresses the candidate small hairpin RNA (shRNA) together with an enhanced green fluorescent protein (EGFP); the other expresses a 19-nt target sequence inserted into a cassette expressing a red fluorescent protein (either DsRed or mCherry). Effectiveness of the candidate shRNA is evaluated as the extent to which it knocks down expression of the red fluorescent protein. Thus, the red-to-green fluorescence intensity ratio (appropriately normalized to controls) is used as the read-out for quantifying the siRNA efficacy at the individual cell level. We tested this dual fluorescence assay and compared predictions to actual endogenous knockdown levels for three different genes (vimentin, lamin A/C and Arp3) and twenty different shRNAs. For each of the genes, our assay successfully predicted the target sequences for effective RNAi. To further facilitate testing of RNAi efficacy, we developed a negative selection marker ( *ccdB*) method for construction of shRNA and red fluorescent reporter plasmids that allowed us to purify these plasmids directly from transformed bacteria without the need for colony selection and DNA sequencing verification.

## Introduction

RNA interference (RNAi) has become as an important tool not only for the identification of gene function but also for therapeutic applications ^1– 3^. RNAi is mediated by small interfering RNA (siRNA), typically 21-nt in length. The siRNA, together with other cytoplasmic protein factors, forms the RNA-induced silencing complex (RISC). The RISC recognizes the target mRNA through base pair matching, and degrades the mRNA by cleavage of the siRNA/mRNA base-matching region. As a commonly used method, a small hairpin RNA (shRNA) is experimentally expressed in cells, after which an endogenous activity (DICER) removes the loop of the hairpin to generate a functional siRNA. As a result, the expression of genes of interest can be specifically silenced ^1^.

One of the biggest technical obstacles in using RNAi techniques is that the efficacy of gene silencing varies among different siRNA molecules ^4– 6^. The difference in efficacy of siRNA molecules is mainly dependent on the first 19-nt sequence of the sense strand of siRNA. Based on systematic analyses of the common features of highly effective siRNAs, several theoretical algorithms have been developed for the design of highly effective siRNA against mRNA targets ^7– 9^. However, the designed sequences often fail to behave as predicted and, therefore, it is still necessary to evaluate the efficacy of candidate siRNAs by experimental methods. Standard methods of evaluating RNAi have been Western blotting, immunofluorescence and quantitative RT-PCR. Alternative experimental approaches using reporter assays based on luciferases or fluorescent proteins have also been developed ^10– 12^. In these reporter assays, a DNA fragment of the gene to be silenced is either inserted into the untranslated region (UTR) of a reporter gene, or connected to the reporter in frame to express a fusion protein. After co-transfection of an siRNA or shRNA expression plasmid with the reporter plasmid, the enzymatic activity of luciferases or the fluorescence intensity of fluorescent proteins is measured. Although both standard and reporter methods have been useful in evaluating RNAi efficacy, a significant limitation is that they are generally applied to populations of cells. If expression of siRNA is not uniform in the population or if only a subset of cells expresses the knockdown construct, population methods of evaluating knockdown will introduce ambiguity into the results. Such ambiguity could be removed if the effectiveness of siRNA could be evaluated in individual cells.

In this article, we report the development of a quantitative, ratiometric reporter assay at the single-cell level. This assay is based on two technical advancements. The first is an improvement in the fluorescence reporter assay itself based on the use of quantitative fluorescence microscopy. Two fluorescence reporter constructs are used. One expresses the candidate shRNA together with a green fluorescent protein (EGFP); the other expresses a 19-nt target sequence inserted into a cassette expressing a red fluorescent protein (either DsRed or mCherry). Effectiveness of the candidate shRNA is evaluated depending on the extent to which it knocks down expression of the red fluorescent protein. Thus, the red-to-green fluorescence intensity ratio (appropriately normalized to controls) is used as the read-out for quantifying siRNA efficacy at the individual cell level. The image acquisition and analysis in our assay is simple and straightforward as only a standard fluorescence microscope system and image analysis software are necessary. The second advance is an improvement in the methods of preparing the required constructs, both the shRNA expression and reporter plasmids. By using a negative selection marker in the transforming plasmid, the *ccdB* gene, virtually all colonies of transformed bacteria contained the correct plasmid constructs. As a result, shRNA expression and red fluorescence reporter plasmids could be prepared without the need for colony selection and DNA sequence verification. We evaluated our dual fluorescence reporter assay using three test genes, vimentin, lamin A/C and Arp3, and confirmed that our fluorescent protein-based reporter assay could successfully predict siRNA efficacy with high fidelity. Taken together, these technical improvements facilitate the selection of highly effective siRNA among multiple candidates and make the ratiometric, fluorescence reporter assay practical and useful at the single-cell level.

## Results

### Design of fluorescence-based knockdown assay

The basic design concept of the assay is to express the candidate shRNA in one construct (the knockdown construct) that also expresses a reference (green) fluorescent protein and to express the target sequence in another construct (the target construct) that also expresses a reporter (red) fluorescent protein. The target sequence is identical to a sequence in the mRNA intended to be knocked down and is thus a proxy for the mRNA. The two types of construct were designed to be as similar as possible so that transfection and expression levels would also be similar.

**Figure 1.**
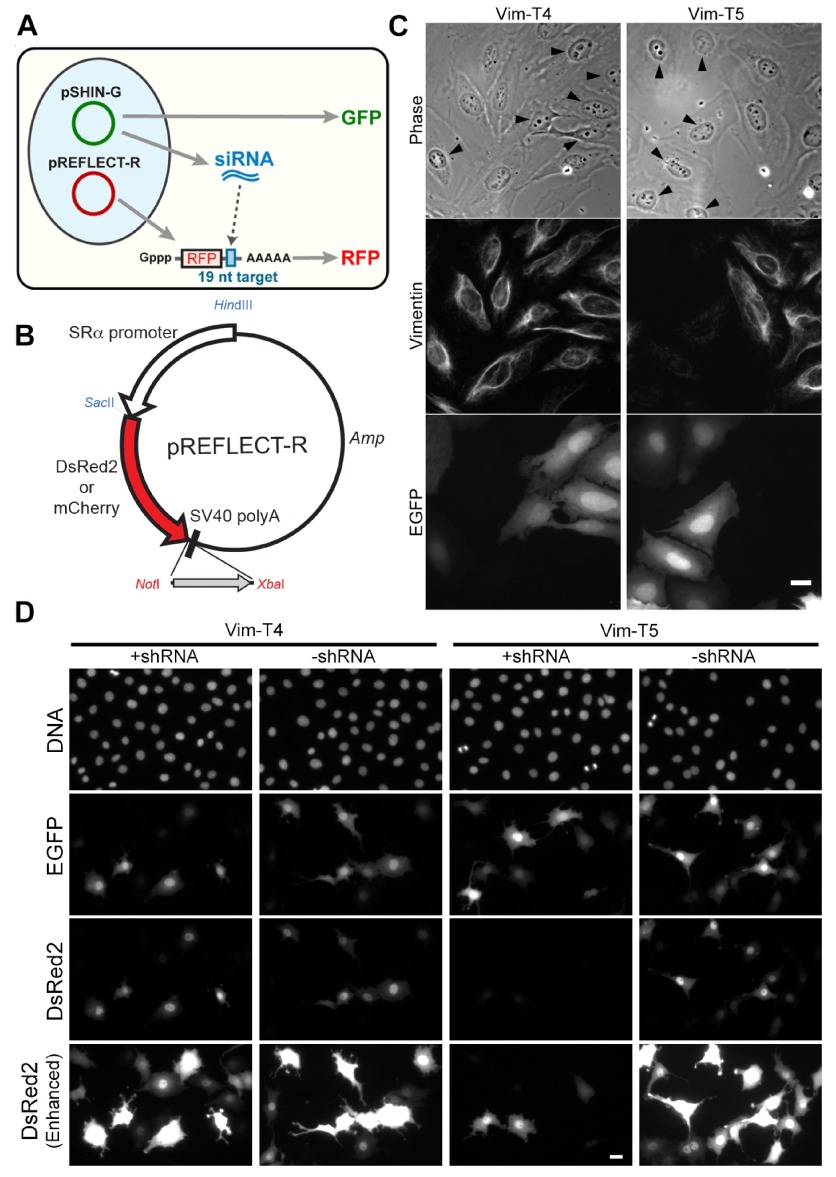
Dual fluorescence ratiometry assay. ( **A**) Experimental design. Two plasmids (pSHIN-G and pREFLECT-R) are used. The plasmid pSHIN-G expresses shRNA and GFP simultaneously, whereas pREFLECT-R expresses a red fluorescent protein (RFP), DsRed2 or mCherry. The 3′-UTR of the RFP mRNA contains a 19-nt sequence target of siRNA to be tested. If siRNA, which is generated from shRNA, is effective, RFP expression is suppressed, resulting in reduction of red fluorescence. ( **B**) The map of pREFLECT-R. RFP expression is driven under the SRα promoter. A 19-nt target sequence is inserted between *Not*I and *Xba*I sites. ( **C**) Immunofluorescence of HeLa cells transfected with pSHIN-G-Vim-T4 and -T5. Phase contrast, vimentin-staining and EGFP images are shown. Arrowheads in the phase contrast images indicate transfected cells as shown by EGFP expression. The Vim-T5 shRNA expression suppressed vimentin significantly, while Vim-T4 did not show the RNAi effect. Bar, 20 µm. ( **D**) Fluorescence images of co-transfection of pSHIN-G and pREFLECT-R(DsR) derivatives. Rat2 cells were transfected with pSHIN-G-Vim-T4 plus pREFLECT-R(DsR)-Vim-T4 (Vim-T4; +shRNA), pSHIN-G empty plasmid vector plus pREFLECT-R(DsR)-Vim-T4 (Vim-T4; -shRNA), pSHIN-G-Vim-T5 plus pREFLECT-R(DsR)-Vim-T5 (Vim-T5; +shRNA) or pSHIN-G empty plasmid vector plus pREFLECT-R(DsR)-Vim-T5 (Vim-T5; -shRNA). Two days after transfection, cells were fixed and DNA-stained with Hoechst33342. DNA, EGFP and DsRed2 fluorescence images are shown. The red fluorescence was not changed by shRNA expression of the Vim-T4 shRNA, whereas the Vim-T5 shRNA reduced red fluorescence significantly. Bar, 20 µm.

**Supplemental Figure 1.**
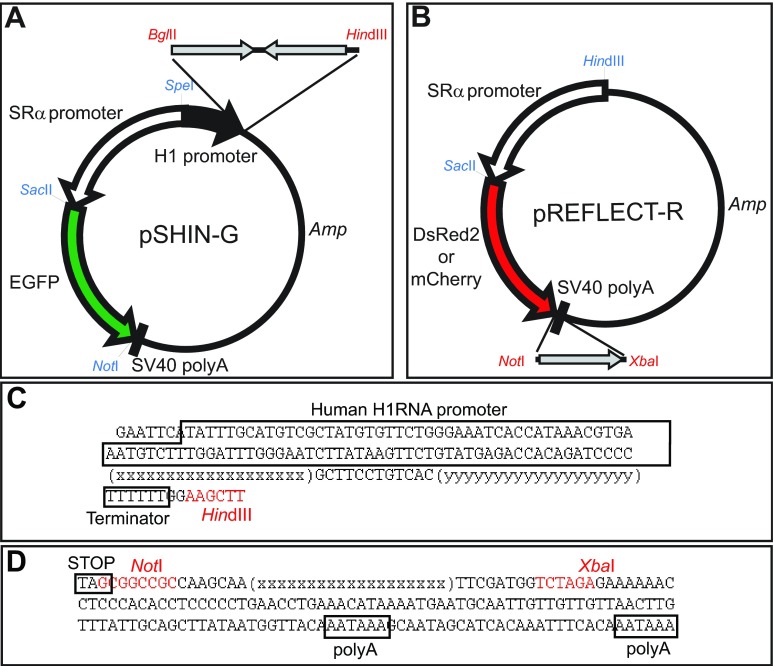
Plasmids: ( **A**) Map of pSHIN-G plasmid. Annealed oligonucleotides including shRNA sequence were inserted between *Bgl*II and *Hin*dIII sites at the downstream of human H1 RNA promoter to make a shRNA expression cassette. EGFP is expressed under SRα promoter for transfection marker. ( **B**) Map of pREFLECT-R plasmid. Annealed oligonucleotides (target sequence) were inserted between *Not*I and *Xba*I sites in the 3′-UTR of RFP (DsRed2 or mCherry). The same SRα promoter and SV40 poly A signal were used between pSHIN-G and pREFLECT-R. ( **C**) Nucleotide sequence of shRNA expression cassette of pSHIN-G derivatives after insertion of oligonucleotides. The xxx… and yyy… represent the sense and antisense strand sequences of the 19-nucleotide target, respectively. Note that oligonucleotide insertion disrupts the *Bgl*II site of the parental vector. Boxes show human H1 RNA and terminator sequences. ( **D**) Nucleotide sequence of the 3′UTR of RFP in pREFLECT-R derivatives after insertion of oligonucleotides. The stop codon (TAG) of RFP and the polyA signals are shown as boxes. The xxx… represents the sense strand sequence of the 19-nucleotide target.

In the target construct (denoted pREFLECT), the 19-nt target sequence is inserted into the 3′ UTR of the reporter DsRed2 or mCherry gene ( Figure 1A, Supplemental Figure 1B). Candidate shRNAs are inserted into the knockdown construct, pSHIN-G expressing EGFP. The experiment consists of co-transfecting the knockdown construct with the target construct and comparing the results to co-transfection of the target construct with the empty pSHIN-G vector. When siRNA works effectively, the reporter mRNA will be degraded by RNAi via the target sequence in the 3′-UTR, resulting in reduction of red fluorescence.

**Supplemental Figure 2.**
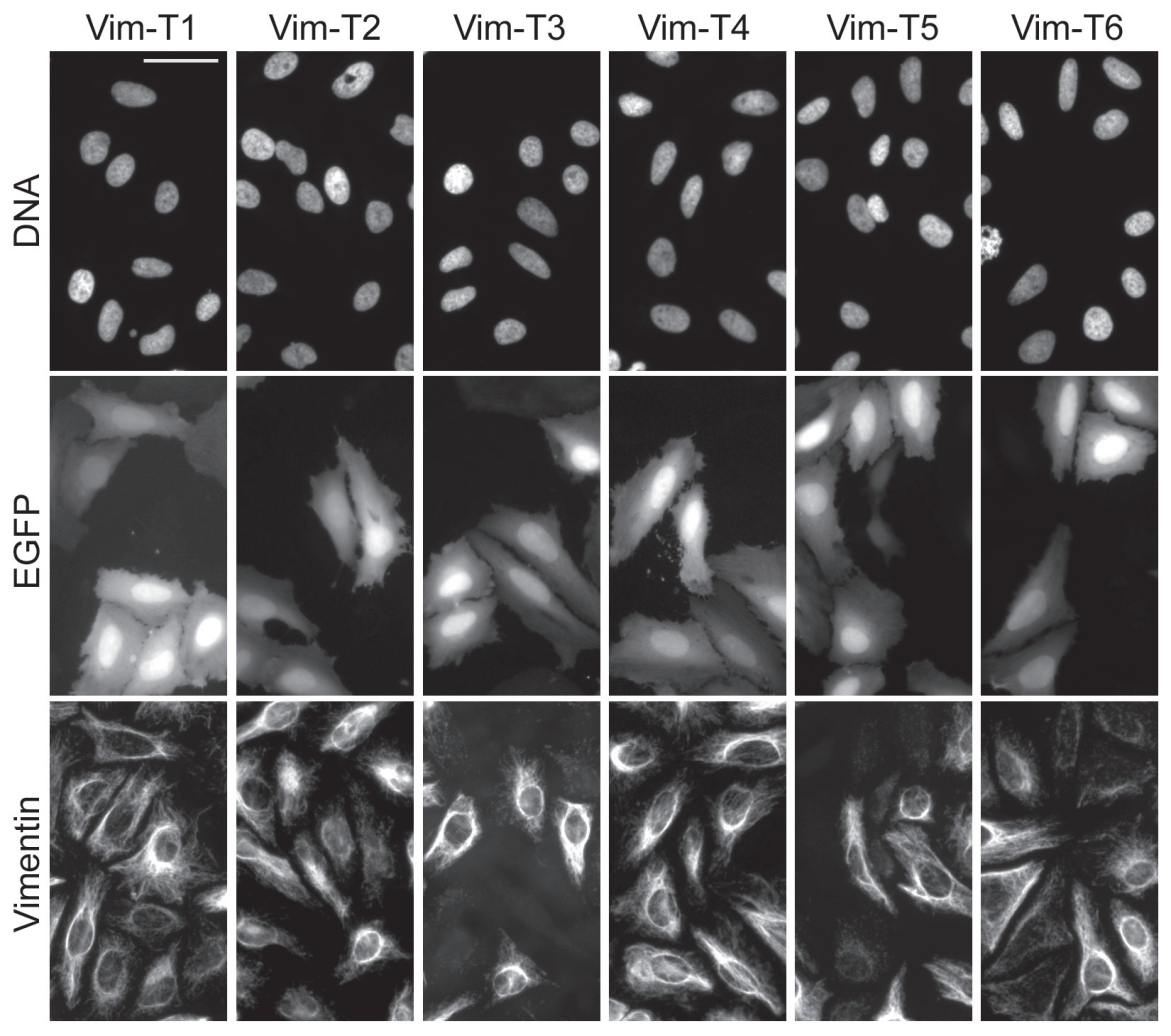
RNAi of vimentin in HeLa cells with six different target sequences. HeLa cells were transfected with each of the pSHIN-G-Vim constructs. Five days after transfection, the cells were fixed and immunostained with mouse monoclonal anti-vimentin antibody. DNA, EGFP and vimentin-staining are shown for Vim-T1 to -T6 shRNA. Different levels of vimentin silencing were observed. Bar, 50 µm.

The initial evaluation of the dual construct system was carried out on the human vimentin gene. We chose six siRNA target sequences (Vim-T1 to -T6; Table 1), previously reported by other groups ^5, 8^. For each target sequence, the shRNA expression plasmids (pSHIN-G-Vim-T1 to -T6) were constructed as reported in the literature ^13^. Five days after transfection of HeLa cells, immunofluorescence revealed that reduction of vimentin differed dependent on the target sequences. Vim-T4 did not show detectable reduction of vimentin ( Figure 1C), whereas reduction was observed for the others, albeit at differing levels ( Supplemental Figure 2). In particular, with Vim-T5, some cells lacked vimentin almost completely (arrowed cells in Figure 1C). The overall results are in good agreement with the previous reports ^5, 8^.

**Table 1.** Target sequences for shRNA validation in this work.

Target name	Sequence (5′ to 3′)	Target gene	Reference
Vim-T1	CTACATCGACAAGGTGCGC	Human vimentin	Nature 411:494–498 (2001) ^4^; J. Cell Sci. 114:4557–4565 (2001) ^5^
Vim-T2	TACCAAGACCTGCTCAATG	Human vimentin	J. Cell Sci. 114:4557–4565 (2001) ^5^
Vim-T3	GAATGGTACAAATCCAAGT	Human vimentin	J. Cell Sci. 114:4557–4565 (2001) ^5^
Vim-T4	ACCAACGACAAAGCCCGCG	Human vimentin	Nucleic Acids Res. 32:936–948 (2004) ^8^
Vim-T5	GTACGTCAGCAATATGAAA	Human vimentin	Nucleic Acids Res. 32:936–948 (2004) ^8^
Vim-T6	GATGAGATTCAGAATATGA	Human vimentin	Nucleic Acids Res. 32:936–948 (2004) ^8^
Lmna-T1	AGCAGTCTCTGTCCTTCGA	Human lamin A/C	Genes Dev. 22:3409–3421 (2008)
Lmna-T2	ACCTGCAGGAGCTCAATGA	Human lamin A/C	This article
Lmna-T3	ACTGAGCACTGCTCTCAGT	Human lamin A/C	This article
Lmna-T4	AGTCTGCTGAGAGGAACAG	Human lamin A/C	This article
Lmna-T5	GCTGCGCAACAAGTCCAAT	Human lamin A/C	This article
Lmna-T6	GCAGATCAAGCGCCAGAAT	Human lamin A/C	This article
Lmna-T7	ACCAGGTGGAGCAGTATAA	Human lamin A/C	This article
Lmna-T8	ATGATCCCTTGCTGACTTA	Human lamin A/C	Genes Dev. 22:3409–3421 (2008)
Arp3-T1	GCAGCTGTATTAAACACAT	Mouse/Rat Arp3	This article
Arp3-T2	ACATTGTCCTCTCTGGTGG	Mouse/Rat Arp3	This article
Arp3-T3	GCCCAAGCCTATTGATGTA	Mouse/Rat Arp3	This article
Arp3-T4	GCCTGAGTTCTACCAAGTA	Mouse/Rat Arp3	This article
Arp3-T5	GGAGTCATGTCCTAAAGTT	Mouse/Rat Arp3	This article
Arp3-T6	GCTTGGATCTAAGAAGCTA	Mouse/Rat Arp3	This article
Ctrl1	GATTTTCGAATCCAAGGCT	Scramble control	This article
Ctrl2	GCGAACCATTTTGTCAACC	Scramble control	This article

**Supplemental Figure 3.**
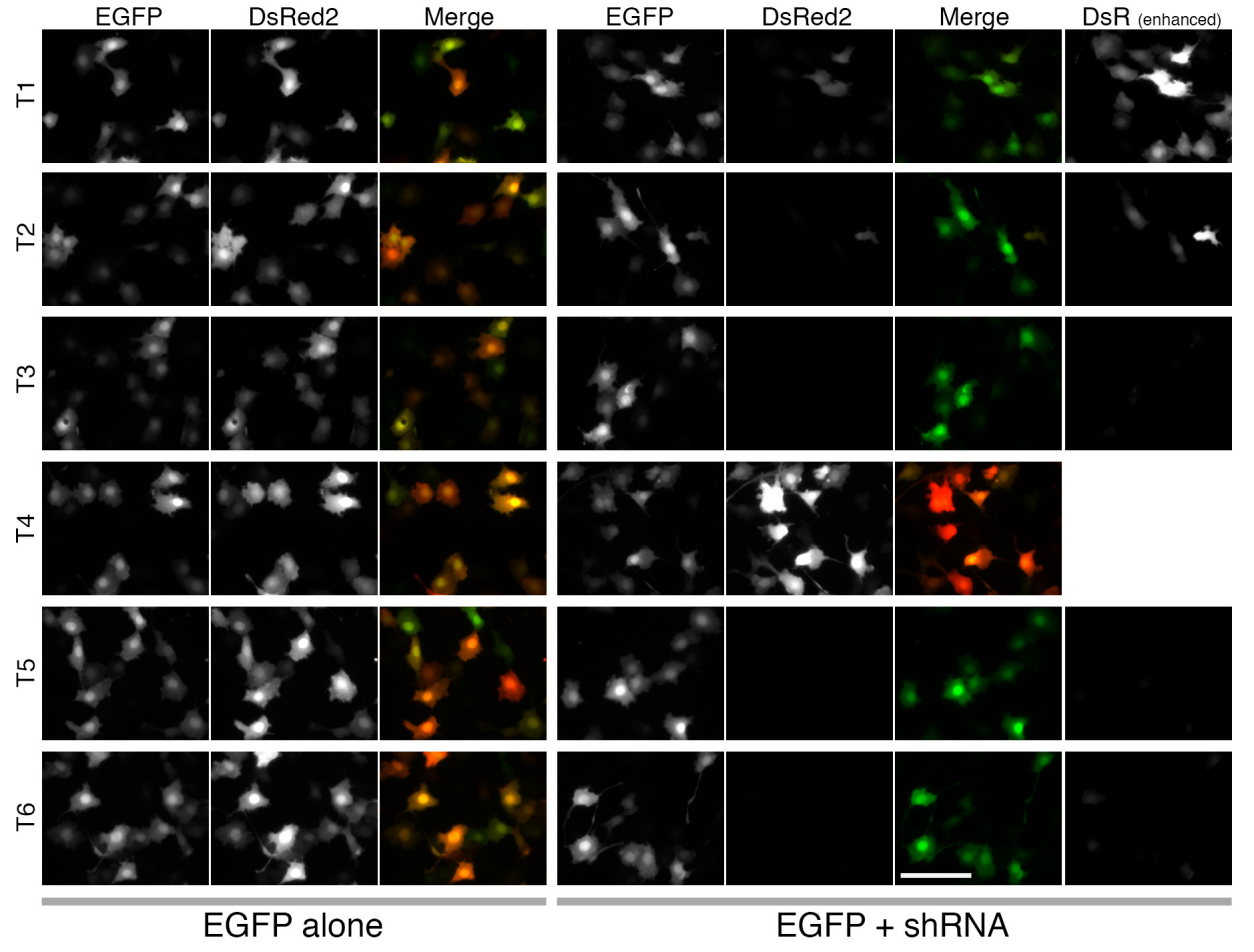
Dual fluorescence assay of vimentin shRNA efficacy. Rat2 cells were transfected with combinations of either pSHIN-G empty vector plus pREFLECT-R(DsR)-Vim (EGFP alone) or pSHIN-G-Vim plus pREFLECT-R(DsR)-Vim (EGFP + shRNA). Two days afterwards, fixed cells were observed by fluorescence microscopy. In the merged images, EGFP and DsRed2 fluorescence are show as green and red colors. The reduction of red fluorescence differed dependent on the target sequences. Bar, 100 µm.

Having confirmed the relative activity of six different shRNAs against endogenous vimentin, we then tested whether the cognate 19-nt target sequences would show the same relative activity. Red fluorescence reporter plasmids were constructed by insertion of annealed oligonucleotides to the 3′-UTR of the DsRed2 red fluorescence protein (pREFLECT-R(DsR)-Vim-T1 to -T6; Figure 1B). Then the reporter plasmid was co-transfected with the corresponding shRNA expression plasmid (test experiment) or the empty pSHIN-G plasmid vector without hairpin sequence (control experiment) at the molar ratio of 4:1. The rat fibroblastic line, Rat2, was used for this assay because the Rat2 cells had nuclei with homogenous morphology, and this morphological feature made image analysis easy (see below). Two days after transfection, the red fluorescence levels of expressed DsRed2 were compared between the test and control samples. As shown in Figure 1D, Vim-T5 reduced red fluorescence remarkably, while for Vm-T4 DsRed2 expression was similar between the test and control samples. The suppression of red fluorescence agreed with the silencing activities of siRNA for endogenous vimentin ( Figure 1D & Supplemental Figure 3). Thus, a simple 19-nt sequence in the context of our fluorescence reporter assay seemed to work, at least qualitatively, as a proxy for mRNA knockdown.

### Image analysis of fluorescence reporter assay

**Figure 2.**
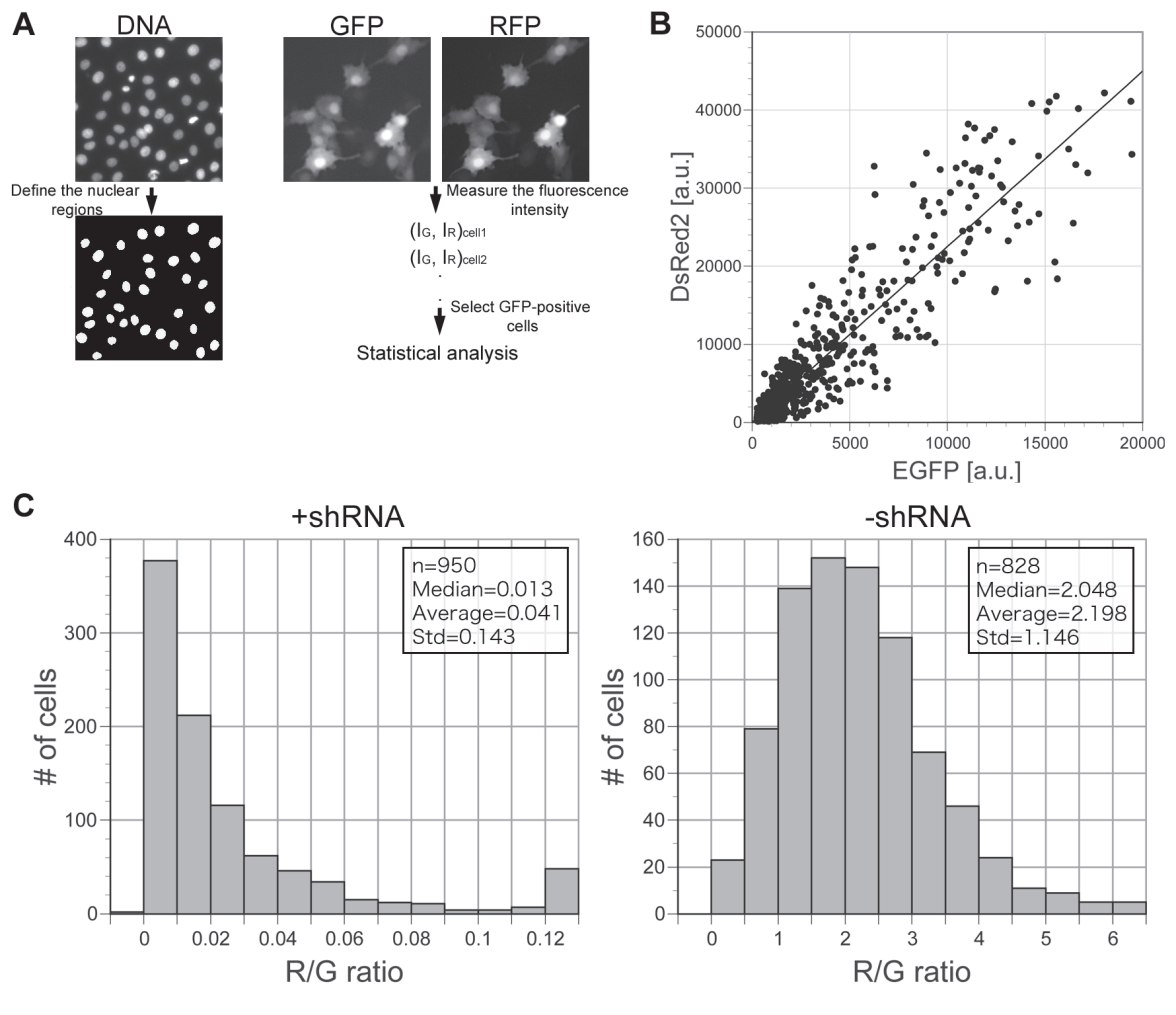
Image analysis of dual fluorescence assay. ( **A**) Scheme of image analysis. Interphase nuclei are defined from DNA-staining images. Although the expressed fluorescence is distributed throughout the cell, regions of interest defined by the DNA staining are convenient for measurement of the GFP and RFP intensities of individual cells. The fluorescence intensities were thresholded, averaged over the region of interest and subjected to statistical analysis (see Materials and Methods in detail). ( **B**) Scatter plot of green and red intensities. Rat2 cells were co-transfected with pSHIN-G empty plus pREFLECT-R(DsR)-Vim-T5. Linear correlation between green and red fluorescence was observed over a wide range of expression levels. ( **C**) Histograms of the ratios of red intensity to the green intensity (R/G). Left, co-transfection of pSHIN-G-Vim-T5 plus pREFLECT-R(DsR)-Vim-T5; right, co-transfection of pSHIN-G empty plus pREFLECT-R(DsR)-Vim-T5. The right histogram was created from the same data set as for Figure 2B. Upon shRNA expression (left histogram), the R/G ratios were significantly reduced because of RNAi of RFP. The medians, averages and standard deviations (Std) of the R/G ratios, as well as the numbers of analyzed cells (n), are shown in the insets.

**Supplemental Figure 4.**
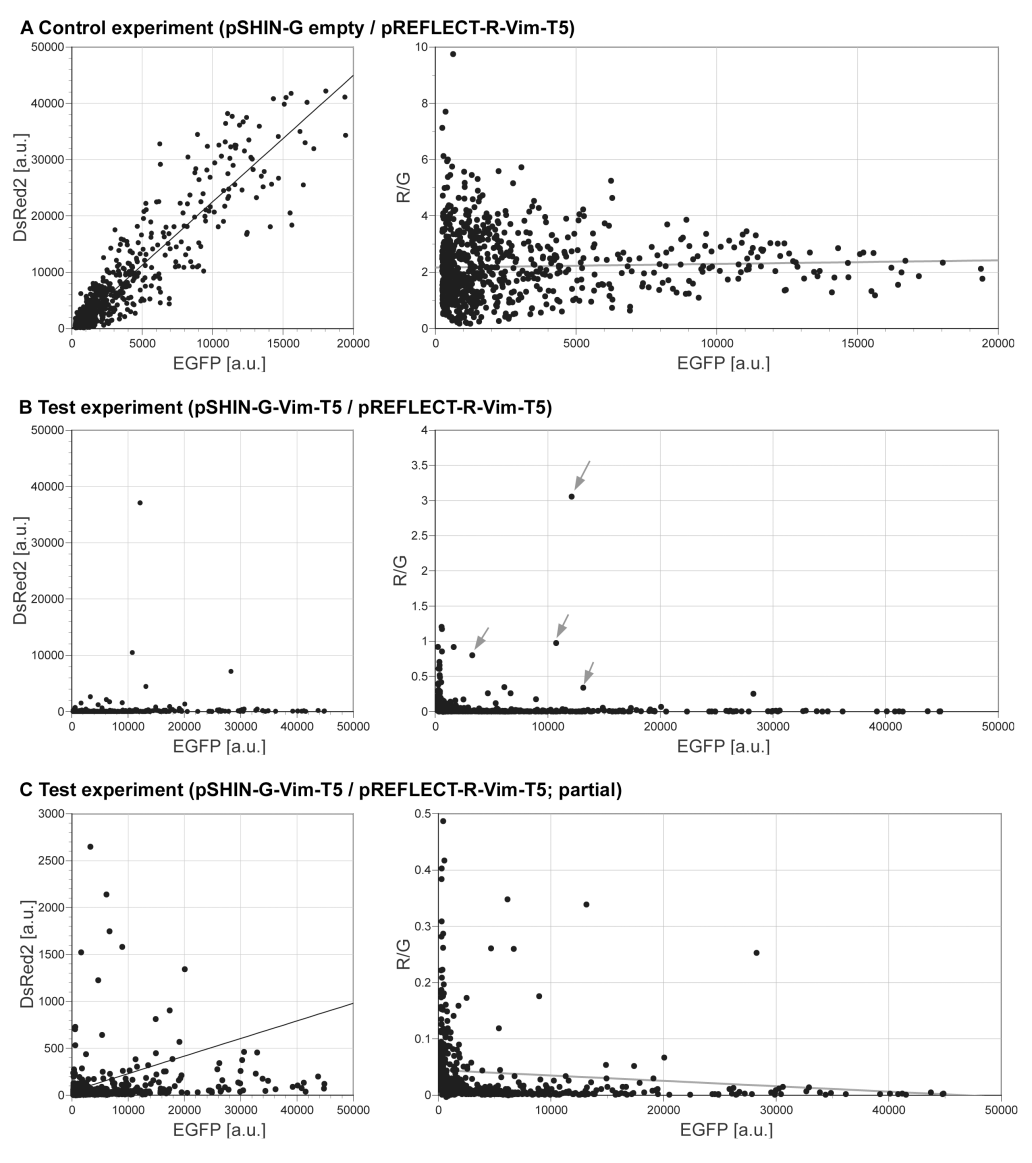
Scatter plots of dual fluorescence assay. Rat2 cells were transfected with combination of either pSHIN-G empty vector plus pREFLECT-R(DsR)-Vim-T5 ( **A**) or pSHIN-G-Vim-T5 plus pREFLECT-R(DsR)-Vim-T5 ( **B**& **C**). The EGFP and DsRed2 fluorescence intensities were measured for individual nuclei of transfectants expressing EGFP. The x-axis indicates the EGFP intensity. The y-axis indicates either the DsRed2 intensity (left) or the R/G ratio (right). The same data set is shown in **B** and **C** with different scales. The arrows indicate examples of the outliers with unusually high red fluorescence.

**Supplemental Figure 5.**
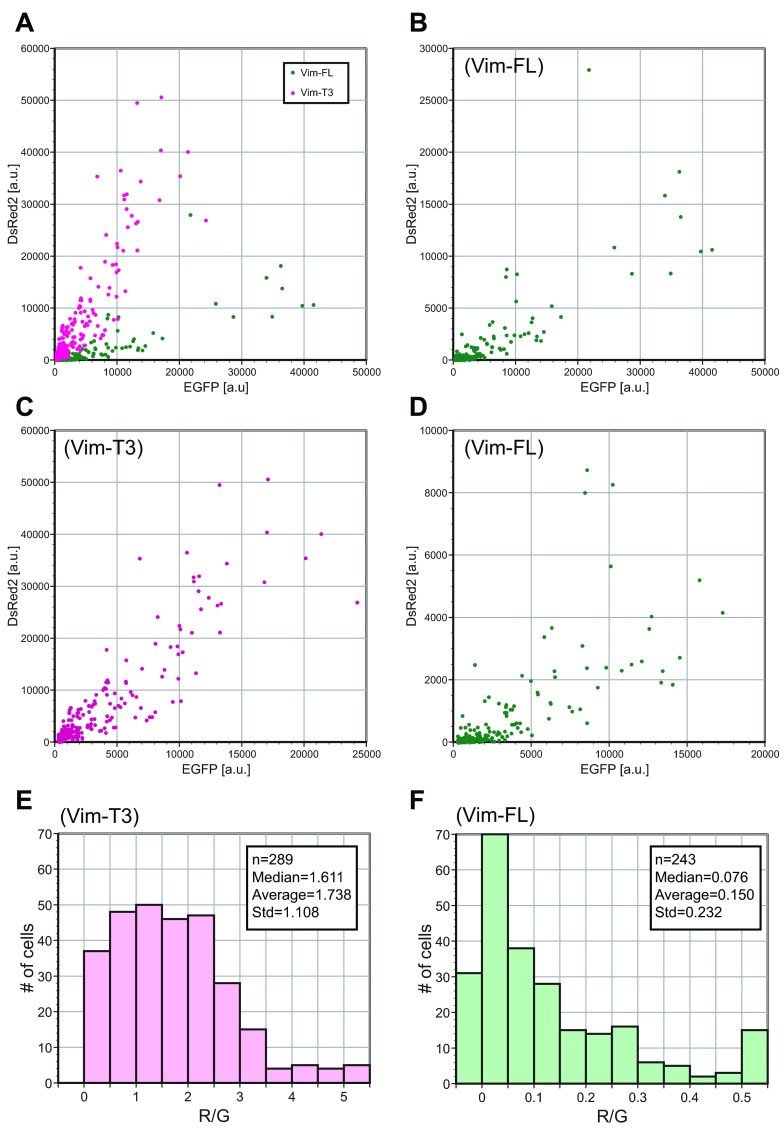
Comparison between full length cDNA and 19-nt in the dual fluorescence assay. Rat2 cells were transfected with either pSHIN-G plus pREFLECT-R(DsR)-Vim-T3 or pSHIN-G plus pREFLECT-R(DsR)-Vim-FL containing the full length coding sequence of human vimentin. Plasmids were mixed at the weight ratio of 1:4. Two days after, cells were imaged and analyzed as in Figure 2. ( **A**) Scatter plot of the Vim-T3 and Vim-FL transfectants. The DsRed2 fluorescence of Vim-FL was lower than that of Vim-T3. ( **B**& **D**) Scatter plots of the Vim-FL transfectants at different scales. ( **C**) Scatter plot of Vim-T3. The correlation between green and red fluorescence of the Vim-T3 transfectants was better than that of the Vim-FL ones. ( **E**& **F**) Histograms of the R/G ratios of Vim-T3 and Vim-FL, respectively. Note that some EGFP-expressing transfectants did not show red fluorescence, resulting in negative R/G ratios in the Vim-FL samples. The standard deviation (Std) relative to the average of the Vim-FL is greater than that of Vim-T3.

Next, we developed a microscopy-based quantification method for the dual fluorescence reporter assay ( Figure 2A). Transfected Rat2 cells were stained with the Hoechst33342 DNA dye to image and define nuclear regions. Although the expressed fluorescence was distributed throughout the cell, quantification of the green and red intensities was easier for the nuclear regions. DNA staining allowed us to define clear-cut regions for fluorescence measurement at individual cell level with assistance of image analysis software. The intensity of green and red fluorescence was recorded for each defined region. The data relative to transfectants were segregated from the data relative to untransfected cells by setting a threshold on the green fluorescence intensity. In control cells, as shown in Figure 2B (& Supplemental Figure 4A), green and red fluorescence intensities were linearly correlated over a wide range of expression levels. This result indicates that red (R)/green (G) ratios at the individual cell level can be used for statistical analysis. The observed linearity was likely related to the similarity of the expression cassettes between pSHIN-G and pREFLECT-R ( Supplemental Figure 1), which use the identical promoter and polyA additional signal, as we could not observe good correlation between green and red fluorescence when EGFP and DsRed2 were expressed under different promoters. The length of the 3′-UTR was also likely important. When the full length of human vimentin coding sequence was inserted into pREFLECT-R(DsR), the expression level of red fluorescent protein was reduced significantly as compared to the 19-nt sequence and the green and red fluorescence intensities did not correlate as well ( Supplemental Figure 5).

**Figure 3.**
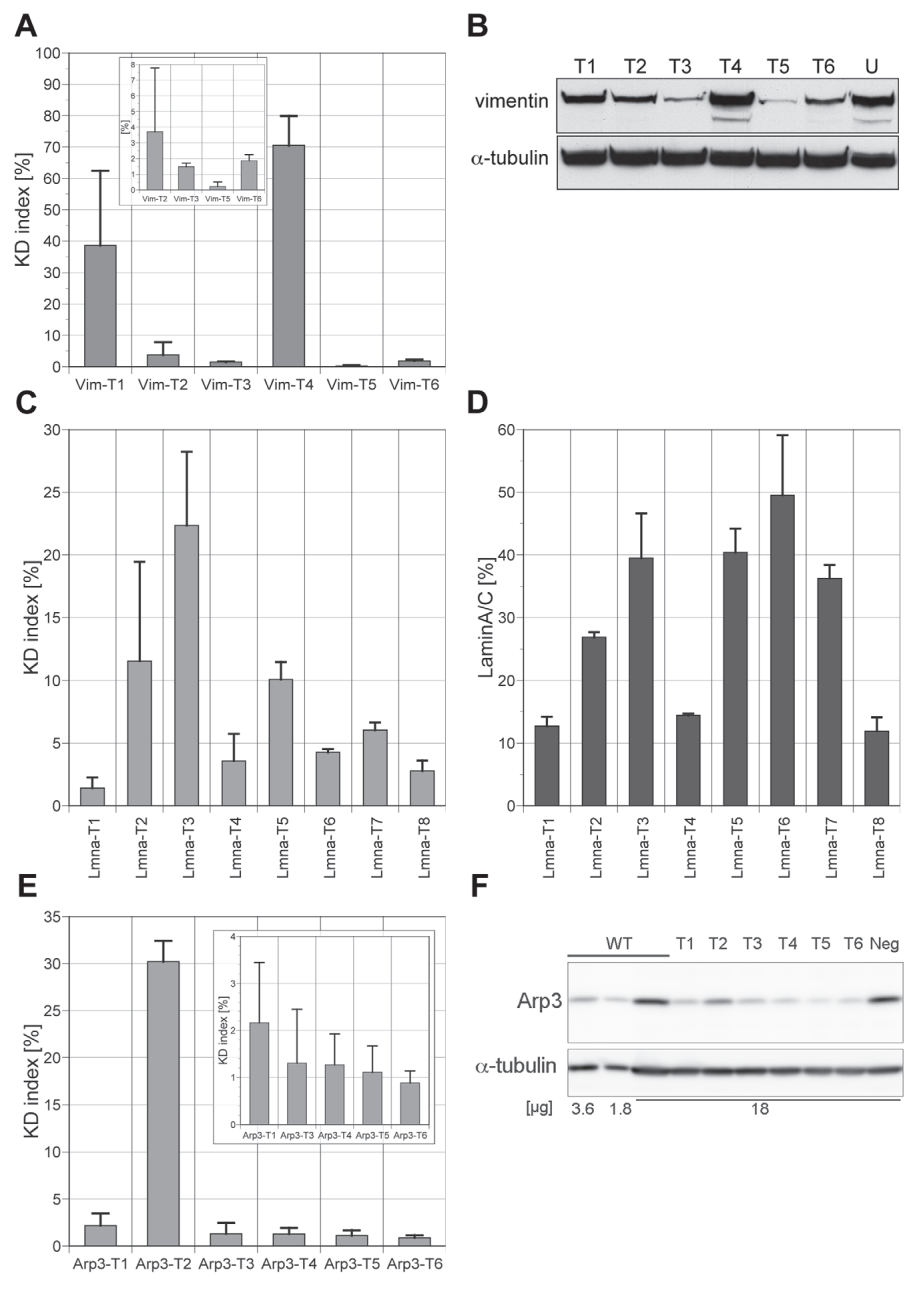
Validation of knockdown **(KD)** indexes in three test systems. The KD indexes were calculated from the medians of the R/G ratios. Bar graphs show the averages and standard deviations of the KD indexes in two or three experiments for each test system. ( **A**, **B**) vimentin; ( **C**, **D**) lamin; ( **E**, **F**), Arp3. ( **A**) KD indexes for Vim-T1 to -T6. The KD indexes of Vim-T2, -T3, -T5 and -T6 are also shown in the inset on an expanded scale. ( **B**) Western blotting of HeLa cells for vimentin. HeLa cells were electroporated with each of the pSHIN-G-Vim constructs. In each lane, 30 µg of the protein sample was loaded. After detection with anti-vimentin antibody, the same membrane was analyzed by anti-α-tubulin antibody (loading control). ( **C**) KD indexes for Lmna-T1 to -T8. ( **D**) Quantification of expression level of laminA/C. The laminA/C expression levels of transfectants as assayed by immunofluorescence were normalized to the averages of untransfected cells. In each experiment, the median of the laminA/C of 50–225 transfected cells was calculated. The averages and standard deviation of two experiments are shown. ( **E**) KD indexes for Arp3-T1 to -T6. The graph in the inset uses an expanded scale for the y-axis. ( **F**) Western blotting of B16S cells for Arp3. B16S cells were transfected with one of pSHIN-puro-Arp3 constructs, and selected in the presence of puromycin for 2 days. For transfected and puromycin-selected cells (Arp3-T1 to -T6, and neg), 18 µg of the protein sample was loaded per lane. For untransfected cells (WT), a dilution series of the protein sample was loaded. The same membrane was analyzed by anti-Arp3 and anti-α-tubulin antibodies (loading control).

In the test experiments using pSHIN-G plasmids with high RNAi efficiency such as Vim-T3 and -T5, the R/G ratios were much lower than those of the control experiments ( Figure 2C and Supplemental Figure 4B&C). As shown Figure 2C, the distribution of the R/G ratios was different from the Gaussian distribution in the test experiments, indicating the median, rather than the average, more suitably represented the population of R/G ratios. In the test experiments, a small number of outliers with unusually bright red fluorescence were constantly observed (arrows in Supplemental Figure 4B), which affected the average of the R/G ratios as compared to their median ( Figure 3C). The outliers imply that RNAi worked much less effectively in a few cells. Interestingly, such RNAi-insensitive cells were also observed in the case of knockdown of endogenous genes ^13, 14^.

We defined the knockdown index (KD index) using the medians of the R/G ratios in the test and control experiments (m _test_ and m _control_, respectively);

        KD = 100 * m _test_/m _control_ * δ

where δ is the correction coefficient introduced to adjust differences in EGFP expression levels between the shRNA expression plasmid and the empty vector. The correction coefficient (δ) was experimentally determined (see Supplementary materials for more detailed explanation) by using pREFLECT-R with a 19-nt scrambled sequence. In most cases, δ was nearly 1, implying that the EGFP expression level was not changed by the insertion of the hairpin sequence under human H1 promoter in pSHIN-G. However, for some target sequences, we found that EGFP expression was reduced by the insertion of the hairpin sequence. The KD index is a measure of the extent to which the target remains after knockdown by the candidate shRNA relative to empty vector control and is expressed in per cent. Thus, a KD of 0% signifies the strongest knockdown (no remaining target) whereas 100% signifies no knockdown.

The KD indexes of Vim-T1 to -T6 were calculated for acquired images ( Figure 3A). Silencing of endogenous vimentin was analyzed by Western blotting of HeLa cell extracts 5 days after transfection of pSHIN-G-Vim plasmids ( Figure 3B). The KD indexes were correlated to the gene silencing of endogenous vimentin. Among the six tested shRNA expression plasmids, Vim-T5 had the lowest KD index and showed the greatest knockdown to endogenous vimentin in Western blotting ( Figure 3B).

### Evaluation of the dual fluorescence assay

For our assay system to be generally useful, it should work on any potential target. To test the generality of the assay, we evaluated silencing of two additional targets, lamin A/C and Arp3, a component of the Arp2/3 complex, as below. For silencing of human lamin A/C, we selected eight new target sequences ( Table 1). Four target sequences (Lmna-T1 to -T4) were chosen simply according to the Tuschl’s criteria ^15^, whereas the computer algorithms provided by Invitrogen ( http://rnaidesigner.invitrogen.com/rnaiexpress) and Dharmacon ( http://dharmacon.com/sidesign/) were used to select Lmna-T5 to -T8 targets. The KD indexes were calculated as shown in Figure 3C. The knockdown levels of endogenous lamin A/C were estimated by analyzing immunofluorescence of HeLa cells 5 days after transfection of pSHIN-G-Lmna plasmids ( Figure 3D). Our previous work showed that analysis of immunofluorescence gave more reproducible and quantitative results about RNAi of laminA/C than Western blotting ^13^. The results of the ratiometry ( Figure 3C) were generally in good agreement with RNAi of endogenous lamin A/C. Seven of the eight tested shRNAs behaved as predicted by dual fluorescence assay. The KD indexes indicated the Lmna-T1 (1.4%) and -T8 (2.8%) as the best two sequences for RNAi. In the knockdown experiments of lamin A/C in HeLa cells, Lmna-T1 and -T8 showed the lowest remaining level of lamin A/C (12.7% and 11.9%, respectively). Only Lmna-T6 showed discordant behavior. The Lmna-T6 shRNA expression gave a low KD index of 4.3% but did not effectively silence endogenous lamin A/C ( Figure 3C). Possibly the presence of a local secondary structure of endogenous lamin A/C mRNA might have restricted the access of the siRNA resulting in poor silencing, as previously seen in other cases ^11^.

**Supplemental Figure 6.**
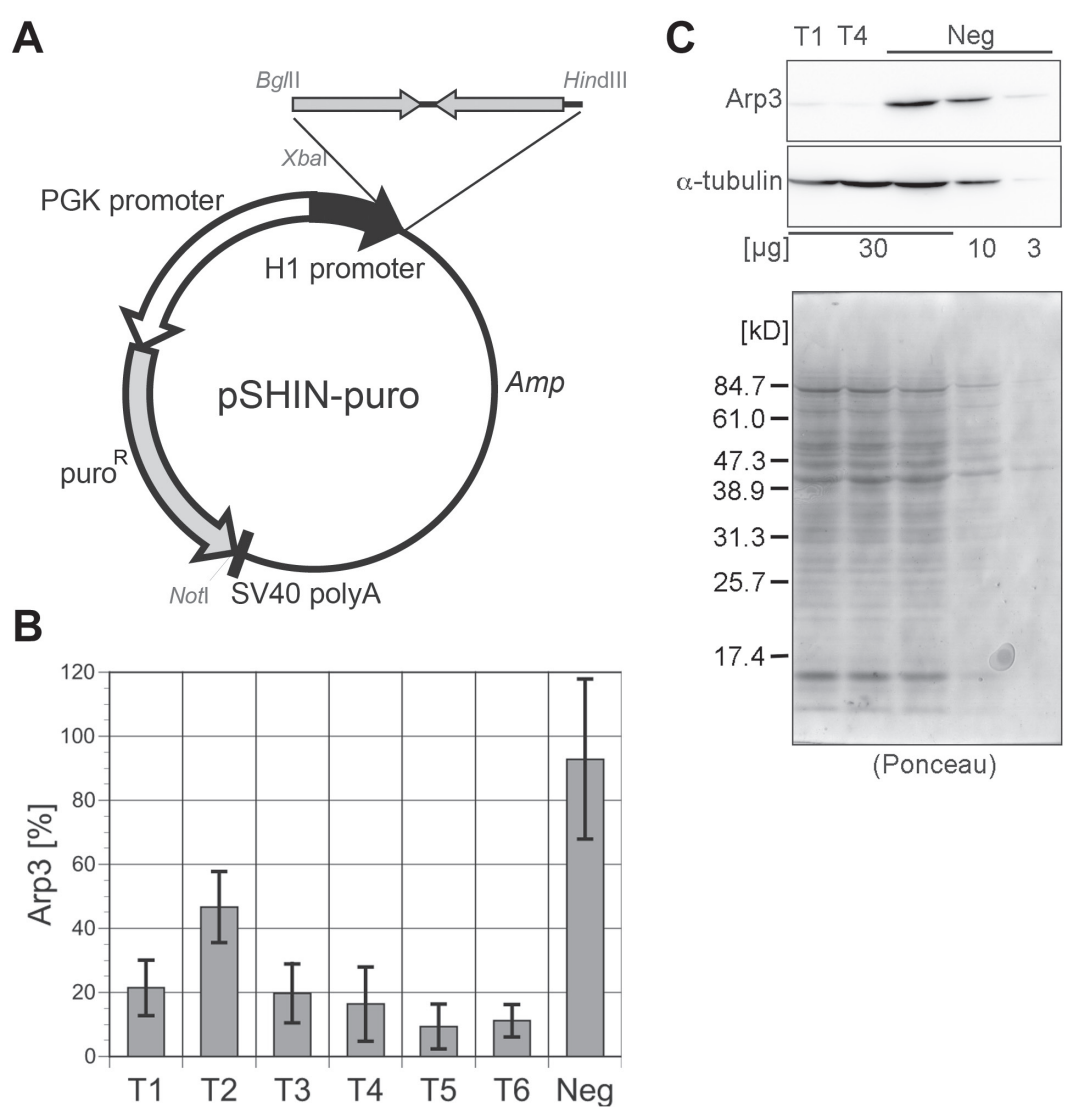
RNAi of Arp3 in B16S and SCC9 cells. ( **A**) The map of pSHIN-puro plasmids. Puromycin resistance gene is expressed under the PGK promoter, whereas shRNA is expressed under human H1 RNA promoter. ( **B**) Quantification of expression level of Arp3 in Rat2 cells. Two independent experiments of Western blotting were analyzed. The remaining protein levels are expressed as percentages of the untransfected Rat2 cells. The averages and standard deviations are shown for RNAi by Arp3-T1 to -T6 and a negative control (Neg) ( **C**) RNAi of human Arp3 with Arp3-T1 and -T4 in comparison to scrambled sequence (negative control, neg). SCC9 cells were transfected with pEpiso-shR[Arp3-T1], [Arp3-T4] or [neg]. Transfectants were selected in the presence of puromycin. The protein extracts were prepared and 30 µg of the T1 and T4 samples were loaded as well as a dilution series (30 µg, 10 µg and 3 µg) of the negative control (Neg) sample. The membrane was analyzed by blotting of rabbit anti-Arp3 antibody and mouse anti-α-tubulin antibodies. Ponceau-S staining of the membrane is shown below the Western blotting. Two independent experiments indicated that Arp3 protein level reduced to 7.8+/-5.1% and 5.1+/-4.6% by Arp3-T1 and -T4, respectively.

In a third test system, we applied our fluorescence reporter assay to select highly effectively siRNA to Arp3. From the common sequence between mouse and rat *Arp3* genes, we picked up six target sequences (Arp3-T1 to -T6; Table 1). We carried out the dual fluorescence reporter assay and the ratiometry image analysis. The resulting KD indexes predicted that five siRNA sequences (Arp3-T1, Arp3-T3 to -T6) would be efficient ( Figure 3E). To confirm endogenous Arp3 knockdown, we constructed shRNA expression plasmids with a puromycin resistance marker (pSHIN-puro-Arp3-T1 to -T6; Supplemental Figure 6A). One day after transfection of pSHIN-puro-Arp3 plasmids to mouse melanoma B16S cells, transfectants were selected in the presence of puromycin for 54 hours. The cell extracts were prepared 5 days after transfection and analyzed by Western blotting. In agreement with the KD indexes, five shRNA expression plasmids except Arp3-T2 reduced endogenous Arp3 proteins to 10–20% level of control cells ( Figure 3F & Supplemental Figure 6B). The best result was obtained with Arp3-T5, which had the second lowest KD index value. As the Arp3-T1 and -T4 sequences are shared with human Arp3 as well, we further examined gene silencing by Arp3-T1 and -T4 in human SCC9 carcinoma cells ( Supplemental Figure 6C). Both constructs silenced Arp3 gene expression significantly as shown in Supplemental Figure 6C for human SCC9 cells.

In summary, our fluorescence reporter assay successfully identified highly effective siRNAs for all three test cases and is likely, therefore, to be generally useful.

### Simplified plasmid preparation

The above results indicate that our dual fluorescence assay is useful to estimate the RNAi efficacy of siRNA. However, the assay requires the construction of appropriate pairs of reporter plasmids which is labor intensive and might limit the general use of this reporter assay. In order to make the dual fluorescence assay more convenient, we modified the procedures of plasmid construction so as to speed up the workflow.

**Supplemental Figure 7.**
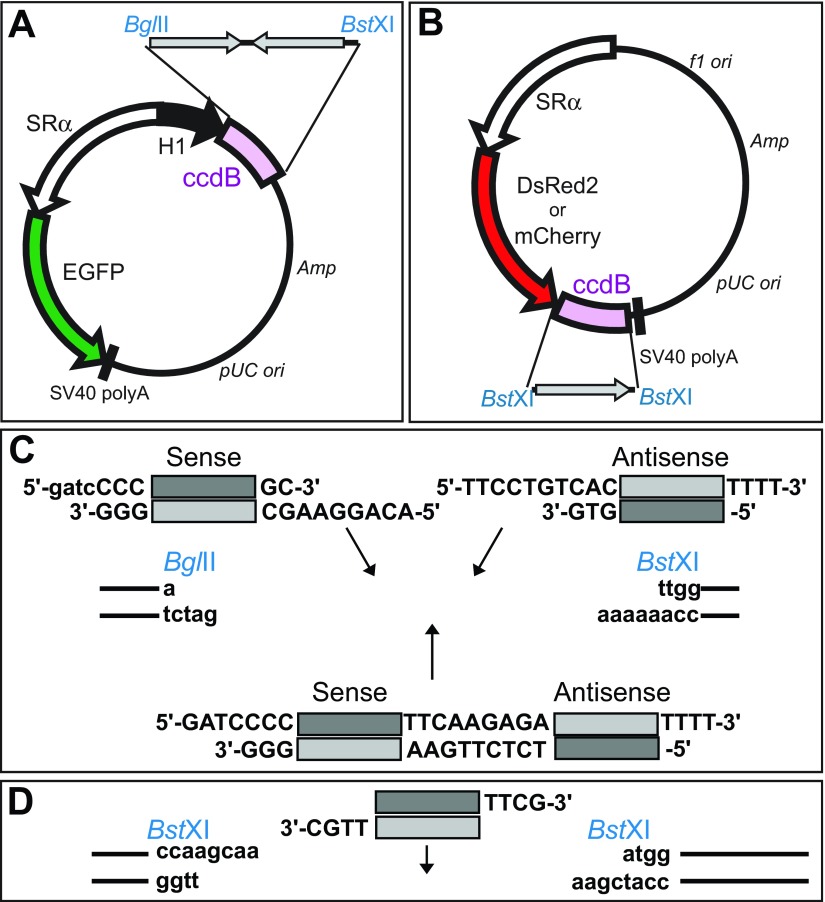
Improved parental plasmid vectors containing the *ccdB* gene as a negative selection marker. ( **A**& **B**) The *ccdB* cDNA was inserted to pSHIN-G ( **A**) and pREFLECT-R ( **B**). Annealed oligonucleotides are inserted into *Bgl*II- *Bst*XI and *Bst*XI- *Bst*XI gaps of pSHIN-G[ *ccdB*] and pREFLECT-R[ *ccdB*], respectively. ( **C**& **D**) Detailed sequence information of the inserted oligonucleotides and the cloning sites is shown.

The key feature of the modification was to incorporate a negative bacterial selection marker. Conventionally, both shRNA expression and fluorescence reporter plasmids are constructed by insertion of annealed oligonucleotides. To obtain the proper plasmid constructs more efficiently, a negative bacterial selection marker, the *ccdB* gene ^16^, was added at the insertion site of oligonucleotides ( Supplemental Figure 7A&B). The *ccdB* gene prohibits these parental vectors (pSHIN-G[ *ccdB*] and pREFLECT-R[ *ccdB*]) to propagate by normal *Escherichia coli* strains. When annealed oligonucleotides are inserted in these vectors to remove the *ccdB* gene, these pSHIN-G and pREFLECT-R derivatives then allow bacterial growth. As a result, virtually all colonies are expected to contain the correct plasmid. Additionally, we used *Bst*XI sites for the oligonucleotide insertion points. As shown in Supplemental Figure 7C&D *Bst*XI-digested overhangs allow unidirectional cloning and prevent insertion of tandems of oligonucleotide duplexes. We tested the success rates of plasmid construction with these modified plasmid vectors. In the case of pREFLECT-R[ *ccdB*], all plasmids contained the correct insert (10 out of 10 colonies). For pSHIN-G[ *ccdB*], we used four short oligonucleotides instead of two long oligonucleotides for plasmid construction as shown in Supplemental Figure 7C. The sense and antisense parts were separately annealed and ligated in tandem to the plasmid vector. Although tandem ligation is thought to be unfavorable, DNA sequencing of purified plasmids revealed that more than 90% of bacterial colonies contained the correct product (13 out of 14 clones). One plasmid contained a 1-nt deletion in the insertion, which was possibly derived from a mistake during DNA oligonucleotide synthesis.

**Figure 4.**
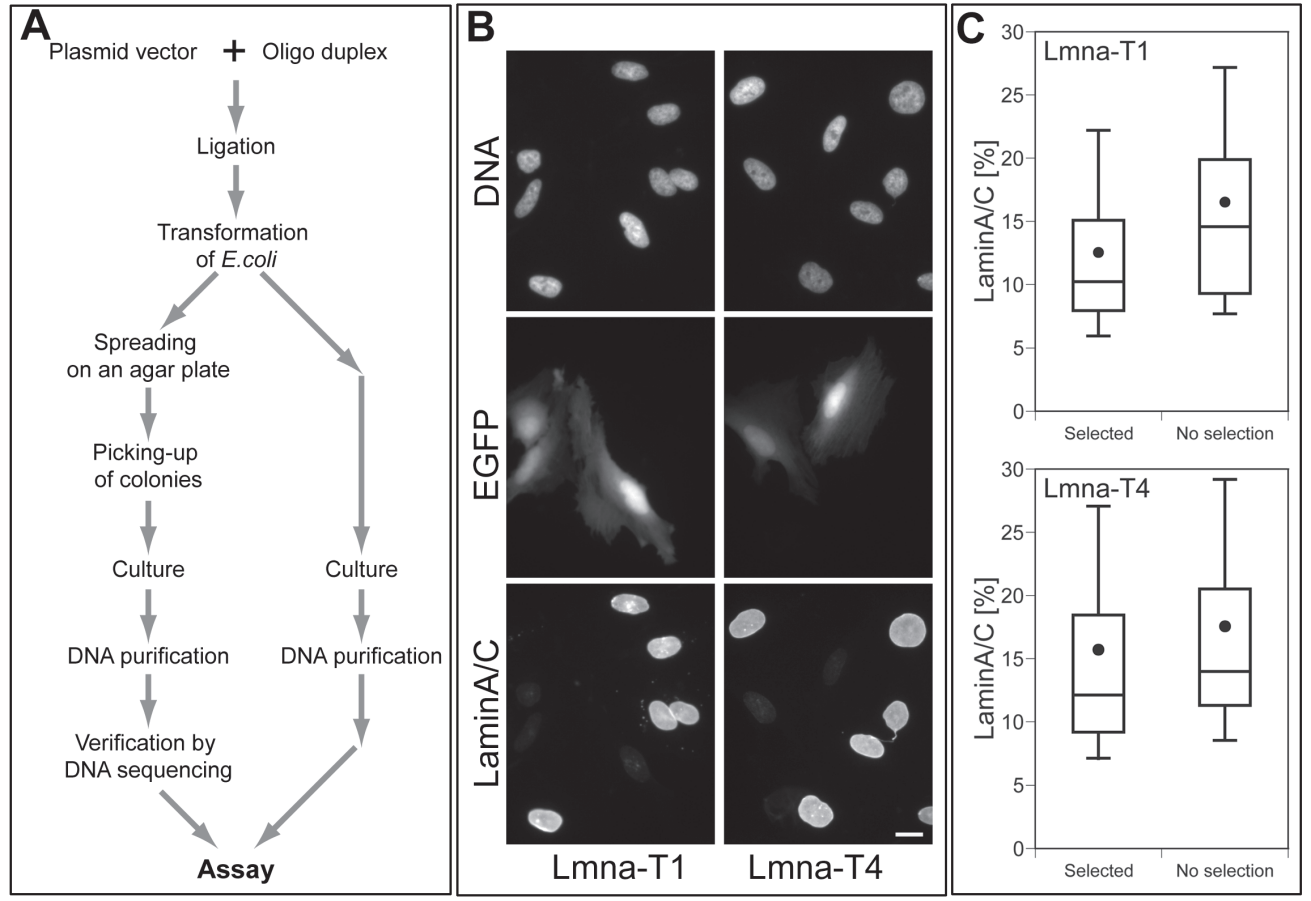
Improved shRNA expression plasmid preparation and demonstration of efficacy. ( **A**) The left flow indicates the conventional method of expression plasmid preparation including colony-selection and DNA sequencing. The right flow indicates the novel method using plasmid vectors with the negative selection marker ( *ccdB*). The *ccdB* gene allows growth of bacteria containing plasmid with insertion of oligonucleotides, but not growth of the parent plasmid vectors. As a result, plasmid DNA can be purified from heterogeneous bacterial culture after transformation without colony-selection and DNA sequencing. ( **B**) Immunofluorescence of lamin A/C in HeLa cells. HeLa cells were transfected with pSHIN-G-Lmna-T1 or -T4 prepared by the “no selection” method (the right flow in **A**). DNA-staining, EGFP and immunofluorescence of lamin A/C are shown. Reduction of lamin staining for cell expressing EGFP indicates knockdown. Bar, 20 µm. ( **C**) Box plots of the lamin A/C levels [%] of HeLa cells transfected with shRNA expression plasmids. The top, bottom, and line through the middle of the box correspond to the 75th percentile (top quartile), 25th percentile (bottom quartile), and 50th percentile (median) respectively. The whiskers indicate the 10th percentile and 90th percentiles. The closed circle represents the mean. Two different methods were used for DNA preparation. The “selected” plasmid DNA was prepared by the conventional method (the left flow in **A**). Preparation of the “no selection” plasmid omits colony-selection and DNA-sequencing as shown in the right flow in **A**. The “no selection” procedure produced results comparable to the conventional procedure. The relative lamin A/C level was calculated as in Figure 3D. In each sample, 129–146 EGFP-expressing cells were analyzed.

**Supplemental Figure 8.**
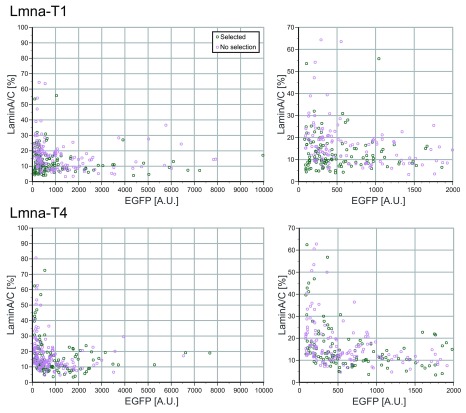
Scatter plots for RNAi of laminA/C. HeLa cells were transfected with pSHIN-G-Lmna-T1 or -T4. Five days after, laminA/C was immunostained. The fluorescence intensity of lamin A/C-staining was normalized to the average of untransfected cells that did not express EGFP. The left and right graphs use different scales for the same data set. The relationship between the EGFP intensities and the remaining lamin A/C levels [%] are shown as scatter plots. The shRNA expression plasmids were prepared by using the two methods described as in Figure 4A. The simplified method without colony-selection (purple dots) was compared with the conventional method (green dots). There was no significant difference between plasmids prepared using the two methods.

Conventionally, DNA clones need to be purified from several bacterial colonies and verified by DNA sequencing (left flow in Figure 4A). However, based on the above results, we hypothesized that plasmid DNA directly purified from transformed *E. coli* would work as well as a construct verified by DNA sequencing (the right flow in Figure 4A). We tested this idea using pSHIN-G-Lmna-T1 and -T4 shRNA expression plasmids that were directly purified from transformed *E. coli* without colony selection. As shown in Figure 4B, lamin A/C expression was efficiently suppressed by these plasmid constructs. We quantified reduction of lamin A/C at the individual cell level ( Figure 4C and Supplemental Figure 8). The silencing was similar for Lmna-T1 and -T4 using both the direct purification method and the conventional plasmid preparation. These results indicate that, for test experiments, the shRNA expression plasmid can be prepared by using our new vector without colony selection.

### Three plasmid system

**Figure 5.**
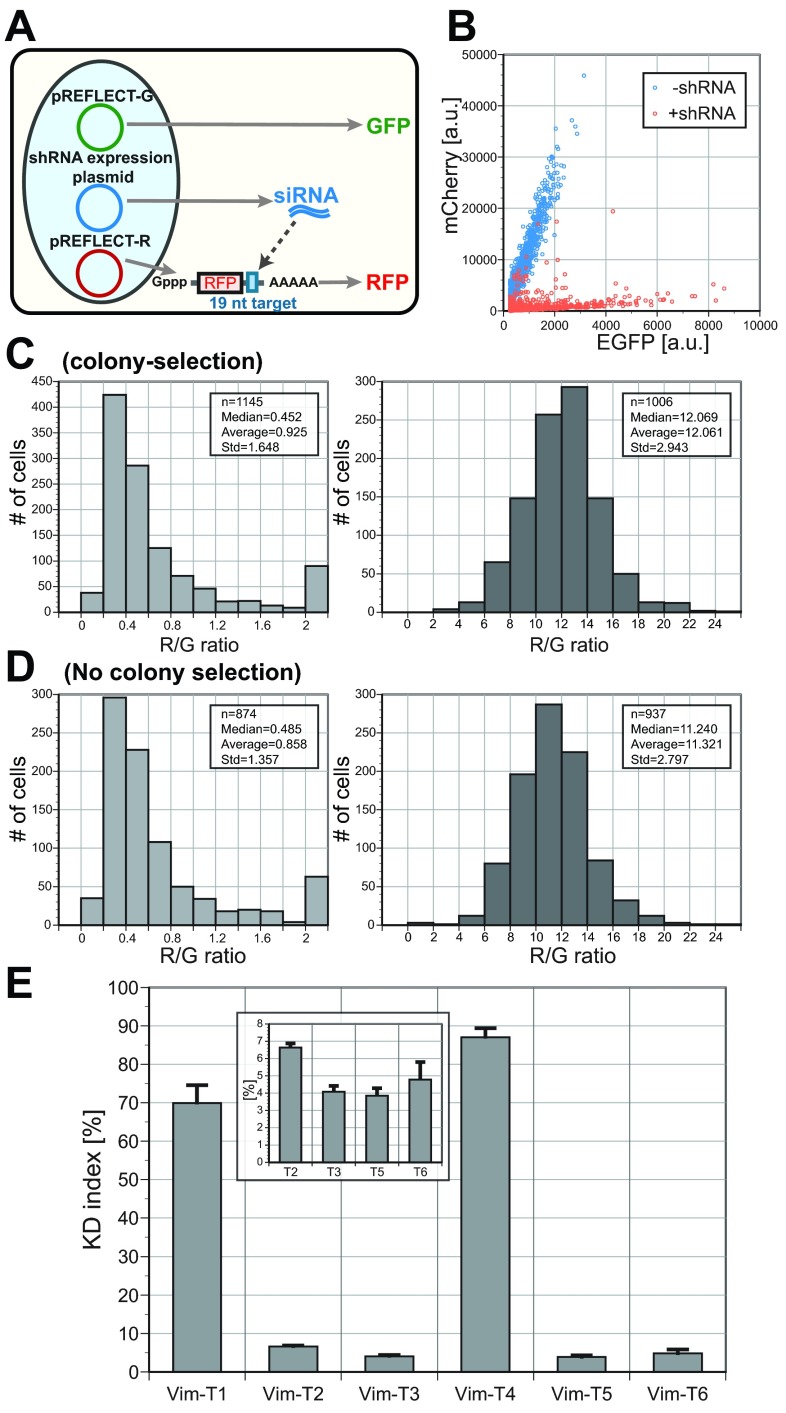
Three plasmid system for dual fluorescence ratiometry. ( **A**) We used three plasmids: the shRNA expression plasmid, the pREFLECT-G and the pREFLECT-R. The pREFLECT-G plasmid expresses GFP as a reference, whereas the pREFLECT-R plasmid expresses RFP as a reporter for RNAi. The 3′-UTRs of the GFP and RFP mRNA contain 19-nt scrambled and target sequences, respectively. ( **B**) Scatter plots of green and red fluorescence intensities. Rat2 cells were transfected with pREFLECT-G-Ctrl-T1 and pREFLECT-R(mCh)-Vim-T3 with or without pSHIN-Vim-T3. The cells were fixed 24 hr afterwards, and the green and red fluorescence intensities were measured. Whereas the green and red fluorescence intensities were both strong and showed linear correlation without shRNA expression (blue dots), the red fluorescence was significantly suppressed by shRNA expression (red dots). ( **C**& **D**) Histograms of the R/G ratios. The results of co-transfection of pREFLECT-G-Ctrl-T1, pREFLECT-R(mCh)-Vim-T3 with and without pSHIN-Vim-T3 are shown (the left and right histograms, respectively). In C, pREFLECT-R(mCh)-Vim-T3 and pSHIN-Vim-T3 were prepared by the conventional method (the left flow in Figure 4A). The no-selection method (the right flow in Figure 4A) was used for both plasmids in **D**. Note expanded scale on left histograms demonstrating efficacy of the 3-plasmids procedure. ( **E**) Knockdown (KD) indexes of Vim-T1 to -T6 target sequences. The bar graph shows the averages and standard deviation of the KD indexes in two independent experiments. Plasmids were prepared by the no-selection method. The results were similar to those obtained by the conventional plasmid procedure as shown in Figure 3. The KD indexes of Vim-T2, -T3, -T5 and -T6 are also shown in the inset.

The original experimental design of the dual fluorescence assay ( Figure 1A) is theoretically ideal as a correlation between EGFP and shRNA is guaranteed by the expression of both the reporter and the hairpin RNA from the same plasmid. However, a drawback of this approach is the need to construct each fluorescent reporter-shRNA plasmid individually. Again, in the interest of speeding workflow and moving towards high throughput, we sought to overcome this drawback using a simplified three-plasmid approach ( Figure 5A). In this approach, the EGFP reporter and the shRNA are expressed on different plasmids with the target carried on a third plasmid as before. With this approach, the EGFP reporter plasmid can be made essentially identical to the target red fluorescent reporter plasmid and the shRNA plasmids can be used without further modification. We tested this system on silencing human vimentin. We used mCherry as the red fluorescence reporter, instead of DsRed2, since mCherry maturates faster than that of DsRed2 ^17^. The red fluorescence became sufficiently bright just 24 hours after transfection of mCherry, whereas DsRed2 required 48 hours. The pREFLECT-G plasmid with a scrambled 19-nt target sequence was constructed by replacing the DsRed2 part of pREFLECT-R(DsR) control plasmid with EGFP. Transfection of the EGFP control and mCherry reporter plasmids to Rat2 cells showed good linear correlation between the green and red fluorescence intensities ( Figure 5B).

As expected, when a shRNA expression plasmid with strong silencing efficacy (Vim-T3) was co-transfected, the red fluorescence intensity level was reduced significantly compared to the green fluorescence intensity level ( Figure 5B). The outliers with unusually bright red fluorescence were not increased significantly compared to the two plasmid assay, implying that three plasmid co-transfection worked efficiently. Next, we tested whether the above simplified plasmid preparation without colony selection was applicable to the dual fluorescence assay using the three plasmids. The plasmid vectors containing the *ccdB* gene (pREFLECT-R(mCh)[ *ccdB*] and pSHIN[ *ccdB*]) were used to prepare mCherry reporter and shRNA expression plasmids, respectively. The distributions of the R/G ratios in the presence or absence of shRNA expression plasmid were similar between the conventional and simplified plasmid preparation ( Figure 5C and D).

The KD indexes were calculated similarly to the original ratiometry assay, using the medians of the R/G ratios (m _test_ and m _control_ for the samples plus and minus the shRNA expression plasmid respectively). In the case of the three plasmid assay, the KD index is simply defined as m _test_/m _control_ without the correction factor. We calculated the KD indexes for six target sequences, Vim-T1 to -T6. The results ( Figure 5E) were similar to those obtained by our original fluorescence reporter assay using two plasmids ( Figure 3A). Therefore the dual fluorescence assay using three plasmids worked as well as the originally developed assay using two plasmids.

## Discussion

We have developed a ratiometric fluorescence reporter assay for siRNA efficacy based on image analysis at the individual cell level. A key advantage of an assay at the single-cell level is that it obviates ambiguities attendant upon methods that average results over populations of cells. With this assay we tested the interaction between various siRNAs and their target sequences in human, mouse and rat cells under the same experimental conditions. Therefore, the results can indicate the intrinsic gene silencing efficacies of different siRNA candidates. Our approach was validated by testing 20 different shRNAs for three different protein systems and, for all shRNAs except one, the estimation by the reporter assay was in good agreement with actual endogenous protein knockdown. The one exception was Lmna-T6. The main difference between our reporter assay and endogenous gene silencing is the surrounding RNA sequences present in the endogenous system. Therefore, we speculate that presence of local secondary structures of lamin A/C mRNA or RNA-binding proteins near the Lmna-T6 sequence might have restricted target recognition by siRNA. It is noteworthy that some candidate sequences (Lmna-T5 and -T7), which were selected by computer algorithms, did not show highly effective gene silencing both in our reporter assay and to endogenous protein. In a series of experiments for three other genes, we first selected five candidate sequences by computer algorithms, but only 1–3 sequences had the KD indexes less than 5% in each case, suggesting that not all candidate siRNAs would have sufficiently strong RNAi efficacy (unpublished data). These results indicate limitations in current theoretical prediction methods and suggest that a realistic RNAi effort still requires testing multiple siRNA targets to validate those that are effective.

Our dual fluorescence assay has the advantage of speeding up and making a more reliable identification of effective siRNAs. We achieved results in only 24 hours, whereas endogenous protein reduction by RNAi usually requires more than 48 hours. In the case of endogenous gene silencing, pre-synthesized proteins are still present when siRNA eliminates the mRNA completely. As a result, it takes a long time before the effects appear at the protein level. The necessary time depends on the protein turn-over rate, but usually 24 hours are not sufficient to reduce the level of endogenous proteins ^4^. In contrast, the synthesis of shRNA and mRNA encoding red fluorescence protein starts simultaneously after transfection in our reporter assay. As a result, red reporter mRNA would be degraded by functional siRNA before red fluorescent protein is translated and accumulated. Thus our assay can predict the siRNA efficacy faster than endogenous gene knockdown experiments. Additionally, this is a good feature for selecting siRNAs whose silencing might be toxic, as the assay will be able to show effective siRNA before the hazardous knockdown phenotypes appear.

We also modified the originally developed two plasmid assay system to accommodate situations where researchers have already invested in existing shRNA expression plasmids. In this situation, the three plasmid system (red reporter, green internal control and existing shRNA) may be of particular utility. Another technical improvement is the simplified construction of shRNA expression plasmids that allow omitting colony selection and sequencing verification. It should be noted that all the steps of simplified plasmid construction, including synthesis and annealing of DNA oligonucleotide, bacterial culture and plasmid DNA purification, are compatible with robotic high throughput platforms. Since our assay is microscopic, automated microscopes for high content screening ^18, 19^ will likely be useful for image acquisition and analysis. Consequently, by this method, preparation of shRNA expression plasmids for high-throughput screening is facilitated. Chemically synthesized siRNA may also be evaluated.

Finally, it should be noted that our microscopic dual reporter assay approach is not limited to siRNA, but could also be applied to other nucleic acid targeting systems. For example, an attractive and possible application of our dual fluorescence assay is the screening of microRNA (miRNA) target sequences. At present, hundreds of miRNAs have been discovered, but their biological functions are poorly understood ^20^. Our assay could easily be applied to miRNA target identification by substituting shRNA expression with miRNA expression.

Recently new genome editing procedures have been developed to modify gene expression. These include transcription activator-like effector nuclease (TALEN) and clustered regulatory interspaced short palindromic repeat (CRISPR)/Cas-based RNA-guided DNA endonucleases ^21^. TALEN and CRISPR/Cas are based on DNA double strand breaks for targeting specific sequences in the genome. Since TALEN and CRISPR/Cas approaches work at the genomic level, they can produce permanent changes in the gene expression as opposed to RNAi which results in transient knockdown of expression. At present, however, several technical obstacles limit TALEN and CRISPR/Cas approaches from general use on culture cells. By both methods, targeting rates were not sufficiently high enough for mammalian culture cells to disrupt both copies of genes ^22– 25^. In addition, off-target genome disruption, as for RNAi, will need to be considered ^26– 30^. Therefore, RNAi is likely to remain useful as a practical technology for suppressing gene expression. In summary, we have developed an image-based, dual fluorescence ratiometric assay to evaluate the knockdown efficacy of siRNA. The assay is microscopic, quantitative and relatively fast. The red fluorescence reporter and shRNA expression plasmids can be directly purified from transformed *E. coli* with or without colony selection and verification by DNA sequencing. A variant three-plasmid procedure allows the use of existing shRNA plasmids.

## Materials and methods

### Reagents

All reagents were purchased from Sigma-Aldrich, unless noting specifically.

### Cell culture

HeLa cells, a human cervical adenocarcinoma cell line, were obtained from the American Tissue Culture Collection (ATCC). HeLa cells were grown in Eagle’s Minimum Essential Medium (MEM) containing Earle’s salts, 2 mM l-glutamine, 0.1 mM non-essential amino acids and 1.0 mM sodium pyruvate, supplemented with 10% fetal bovine serum (FBS). B16S mouse melanoma, Rat2 rat fibroblast and SCC9 human carcinoma lines were provided by Drs. V. Gelfand (Northwestern Univ.), F. Gertler (MIT) and K. Green (Northwestern Univ.), respectively. B16S and Rat2 cells were maintained in Dulbecco’s Modified Eagle Medium (DMEM) supplemented with 10% FBS. SCC9 cells were maintained in Dulbecco’s Modified Eagle Medium: Ham’s Nutrient Mixture F-12 (DMEM/F12, 1:1) media supplemented with 10% fetal bovine serum. All the cell lines were cultured at 37°C with 5% CO _2_. Culture media and FBS were purchased from Gibco and Atlanta Biologicals, respectively.

### Plasmid construction

The parental plasmid vectors, pSHIN-G[ *ccdB*], pREFLECT-R(DsR)[ *ccdB*] and pREFLECT-R(mCh)[ *ccdB*], were constructed as described in detail in the Supplementary materials. These plasmids containing the *ccdB* gene were amplified in a special *E. coli* strain, DB3.1 (Invitrogen). A shRNA expression vector without fluorescent protein reporter, pSHIN[ *ccdB*], was constructed by *Bam*HI digestion and subsequent self-ligation of pSHIN-G[ *ccdB*].

Knockdown constructs except for a negative control were constructed by using sets of four oligonucleotides that were obtained from Integrated DNA Technologies (IDT) at the desalting grade. The construction scheme was illustrated in Supplemental Figure 7C. The oligonucleotides had the following configurations:

#1-fwd: 5′-GATCCCC xxxGC-3′,

#1-rev: 5′-ACAGGAAGC yyyGGG-3′

#2-fwd: 5′-TTCCTGTCAC yyyTTTT-3′

#2-rev: 5′- xxxGTG-3′

where xxx and yyy represent a selected 19-nt target and its complementary sequence, respectively. The loop sequence (GCTTCCTGTCAC) comes from the human *miR23* gene. Oligonucleotides #1-fwd and #1-rev were annealed in 30 mM HEPES, 100 mM K-acetate and 2 mM Mg-acetate (pH7.0) by heating at 95°C for 4 min, incubating at 70°C for 10 min and gradually cooling down to 4°C to make oligoduplex #1. Similarly oligoduplex #2 was prepared. Then the two duplexes were mixed and phosphorylated by T4 polynucleotide kinase in the presence of 0.1 mM ATP at 37°C for 30 minutes, followed by the reaction termination at 70°C for 10 minutes. For a negative control shRNA expression plasmid, two oligonucleotides,

5′-GATCCCC ATGTACTGCGCGTGGAGACTTCAAGAGA GTCTCCACGCGCAGTACATTTTT-3′

5′- ATGTACTGCGCGTGGAGACTCTCTTGAA GTCTCCACGCGCAGTACATGGG-3′,

were annealed and phosphorylated as above. The phosphorylated oligos were ligated with *Bgl*II- *Bst*XI digested pSHIN-G[ *ccdB*] or pSHIN[ *ccdB*] to create shRNA expression plasmids with or without EGFP marker, respectively.

Reporter constructs were constructed by insertion of two oligonucleotides: 5′-xxxTTCG-3′ and 5′-yyyTTGC-3′, where xxx and yyy represent the sense and antisense sequences of a selected 19-nt target respectively ( Supplemental Figure 7D). A pair of oligonucleotides were annealed as above and ligated to a *Bst*XI gap of pREFELECT plasmids. Neither phosphorylation of oligonucleotides nor dephosphorylation of the plasmid vector was necessary. All the target sequences in this experiment are summarized in Table 1.

Knockdown plasmids with puromycin resistant markers were constructed as below. The phosphoglycerate kinase I (PGK) promoter and puromycin resistant (puro ^R^) gene sequences were PCR-amplified from p *Silencer*™ 5.1-H1 Retro (Ambion) with oligonucleotide primers, 5′-AAATCTAGATACCGGGTAGGGGAGGCGCT-3′ and 5′-AAAGCGGCCGCTCAGGCACCGGGCTTGCGG-3′. After digestion with *Xba*I and *Not*I, the amplified fragment was inserted to an *Xba*I- *Not*I gap of pSHIN-G to create pSHIN-puro, which does not contain retroviral sequences. The pSHIN-puro-Arp3 plasmids were constructed by replacement of the expression cassette of EGFP with the one of puro ^R^.

Stable knockdown plasmids for human cells were constructed as below. A *Bsp*HI DNA fragment containing pUC ori sequence was excised from pBluescript KS(+) (from Dr. F. Hanaoka, Gakushuin Univ.), while pLZRS-linker (from Dr. K. Green, Northwestern Univ.) was digested with *Bsp*HI to obtain a DNA fragment containing the oriP and EBNA-1 sequences as well as the puromycin resistant gene. These fragments were ligated to make pEpiso-KS(+). The shRNA expression cassettes were excised by *Bam*HI- *Hin*dIII digestion from pSHIN-G-Arp3-T1, -Arp3-T4 and -neg, and inserted to a *Bam*HI- *Hin*dIII gap of pEpiso-KS(+) to create pEpiso-shR[Arp3-T1], [Arp3-T4] and [neg].

Small scale plasmid DNA was purified from 1.5–2 mL bacterial culture with QIAprep Spin Miniprep kit (QIAGEN) or Perfectprep Plasmid Mini kit (Eppendorf). When using the Perfectprep Plasmid Mini kit, the TritonX-114 extraction step was added for removal of endotoxin. Briefly, after centrifugation of the alkali lysis extract, 3 µL of TritonX-114 was added to ~300 µL of the supernatant. The mixture was cooled on ice for 10 minutes, subsequently incubated at 55° C for 10 min and centrifuged for 5 minutes. The supernatant was used for the following DNA purification with 450 µL of the DNA binding matrix. By this modification, transfection efficiency to Rat2 cells was greatly improved. Competent cells of *E. coli* strains, TOP10 (Invitrogen) and XL10-Gold (Stratagene) with high competency (~10 ^9^/µg) were used. Maxi-prep of plasmid DNA was carried out by using the EndoFree Plasmid Maxi kit (QIAGEN). All the clones were confirmed by DNA sequencing.

### Dual fluorescence assay

Rat2 cells, plated in 12 well plates, were co-transfected with a combination of plasmids by using the transfection reagent TransIT-LT1 (Mirus). For two-plasmid assays, 0.2 µg of a pSHIN-G derivative and 0.8 µg of a pREFLECT-R derivative were used. For three-plasmid assays, 0.2 µg of pSHIN derivative, 0.2 µg of pREFLECT-G-Ctrl and 0.8 µg of pREFLECT-R(mCh) derivative were transfected. After 3–4 hours of incubation with the plasmid DNA/transfection reagent mixture, the cell culture medium was replaced in order to minimize cytotoxicity by endotoxin. The cells were fixed with 4% formaldehyde for 30 minutes at the time point of 48 hour (for DsRed2) or 24 hour (for mCherry) after adding the plasmid DNA/transfection reagent mixture. The fixed samples were permeabilized with 1% TritonX-100 for 5 minutes and DNA-stained with 10 µg/mL Hoechst 33342 (Invitrogen) for 20 minutes. The samples were prepared either on glass coverslips or in plastic culture plates. Fluorescence microscopy was carried out using a DIAPHOTO 300 microscope (NIKON) equipped with a 10× dry objective lens (Plan 10, N.A.0.25, Ph1 DL; NIKON), a CCD camera (CH350; Photometrics) and a filter wheel system (LAMBDA 10-2; Sutter Instrument). For EGFP/DsRed2 imaging, the 86100bs quad filter (Chroma) was used with combinations of excitation/emission filters including D360/40× and S457/50m for DNA, S480/25× and S520/40m for EGFP, and S555/28× and S617/73m for DsRed2. For EGFP/mCherry imaging, the 51019bs EGFP/DsRed dual filter (Chroma) was used with combinations of D360/40× and S457/50m for DNA, S480/25× and S520/40m for EGFP, and S573/23× and S630/75m for mCherry. MetaMorph software (Molecular Devices) was used for image acquisition and analysis. For statistical analysis, Excel (Microsoft) and DeltaGraph (Redrock) software programs were used.

Image analysis was as follow. From DNA-staining images, cellular nuclei were defined. The threshold value was determined manually as approximately 95% of DNA-stained regions had the fluorescence intensity over that value. The continuous regions with the fluorescence higher than the threshold value were recorded by MetaMorph software. Defined regions were ellipses corresponding to cellular nuclei. The defined regions were further filtered for the normal range of nuclear sizes (1.1 µm ^2^–2.7 µm ^2^) and shapes of Rat2 cells and served as regions of interest for further analysis. The average intensities in green (EGFP) and red (DsRed2 or mCherry) channels were recorded for each defined region of interest. The green and red intensities were calculated by subtraction of the backgrounds that were estimated using an untransfected sample of Rat2 cells. The transfectants were selected by thresholding the EGFP intensity. The threshold value was preset from background analysis of pilot experiments under identical imaging condition (see Supplementary materials). Cellular nuclei with EGFP levels above the threshold level were used for further analysis. The ratio of red to green signal (R/G) was calculated for each transfectant. Usually more than 300 data points were collected for each sample, and used to calculate the median. The knockdown index (KD) was calculated by comparison of the median R/G values as described in the Results section and the Supplementary materials.

### Immunofluorescence

HeLa cells were transfected with pSHIN-G-Lmna or -Vim plasmids using the TransIT-LT1 reagent (Mirus) according to the manufacturer’s instruction. Five or six days after adding the plasmid/transfection reagent mixture, the cells were fixed with 4% formaldehyde for 30 minutes and permeabilized with 1% TritonX-100 for 5 minutes. Mouse monoclonal anti-laminA/C (clone 636, Santa Cruz Biotechnology, #sc-7292) or anti-vimentin (clone V9, Chemicon, #MAB3400) antibodies were added to the cells at 1:100 for 30 minutes. After washing with phosphate buffer saline (PBS), the samples were incubated with 10 µg/mL tetramethylrhodamine-conjugated donkey anti-mouse IgG (Jackson Immuno Research, #715-025-150) and 10 µg/mL Hoechst33258 (Invitrogen) for 20 minutes. Images were taken by the above microscope setting except for using 20× objective lens (Fluor 20, N.A.0.75, Ph3DL; NIKON). Endogenous lamin A/C was quantified as previously described ^13^. Briefly, the red fluorescence of lamin A/C-staining was measured and normalized to the average intensity of untransfected cells that did not express EGFP. In each experiment, 50–225 EGFP-expressing cells were analyzed.

### Western blotting

For knockdown of vimentin, HeLa cells were electroporated with pSHIN-G-vim plasmids as described elsewhere ^13^. EGFP-expressing cells usually exceeded 75% of total cells. Five days after electroporation, the protein samples were prepared. For knockdown of Arp3 in B16S cells, pSHIN-puro-Arp3 plasmids were transfected with TransIT-LT1 (Mirus). One day after addition of the DNA/liposome mixture, the cells were cultured in DMEM with 10% FBS and 2.0 µg/mL puromycin for 9 hours, following additional 45 hours culture in the presence of 1.5 µg/mL puromycin. The selected B16S cells were cultured two additional days without puromycin. For knockdown of Arp3 in SCC9 cells, pEpiso-shR[Arp3-T1], [Arp3-T4] or [neg] were transfected using TransIT-LT1 (Mirus). One day after transfection, puromycin was added at the final concentration of 0.8 µg/mL. The transfectants were selected in the presence of 0.8 µg/mL of puromycin for 9 days. Preparation of protein samples, SDS-PAGE, and transferring to PROTRAN nitrocellulose membrane (Whatman) were described elsewhere ^13^. Mouse monoclonal anti-vimentin (clone V9, Chemicon, #MAB3400, 1:1000), rabbit polyclonal anti-Arp3 (Upstate, #07-272 1:500) and mouse monoclonal anti-α-tubulin (clone B-5-1-2, SIGMA-ALDRICH, #T5168, 1:5000) antibodies were used. Horseradish peroxidase (HRP)-conjugated secondary antibodies were obtained from KPL and GE Healthcare. ECL was carried out using ECL Western Blotting Detection Reagent (GE Healthcare), SuperSignal West Pico Chemiluminescent Substrate (PIERCE) or Immobilon Western Chemiluminescent HRP Substrate (Millipore). The chemiluminescent signal was recorded by the LAS-3000 imager (Fuji Film) or detected using Hyperfilm ECL (GE Healthcare). The results were analyzed by ImageJ software.

## Data availability

ZENODO: Data of quantitative, ratiometric dual fluorescence reporter assay at the single-cell level, doi: 10.5281/zenodo.8296 ^31^

## Figures and Tables

**Supplemental Figure 9.**
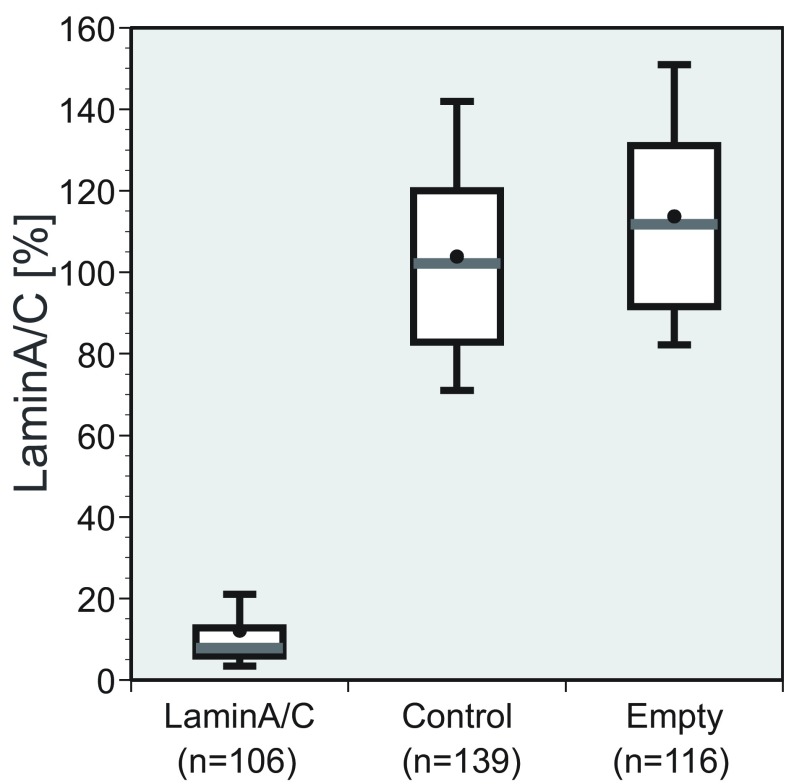
Quantification of laminA/C by immunofluorescence. Immunofluorescence-based quantification of laminA/C was checked for specificity with two different negative controls. The results in our previous work ^13^ are presented by box plots of the lamin A/C levels [%] of untransfected HeLa cells. The top, bottom, and line through the middle of the box correspond to the 75th percentile (top quartile), 25th percentile (bottom quartile), and 50th percentile (median) respectively. The whiskers indicate the 10th percentile and 90th percentiles. The closed circle represents the mean. HeLa cells were transfected with pG-SUPER-hLaminA/C (LaminA/C), -mFascin1 (control) and empty vector not expressing shRNA (empty). pG-SUPER is almost identical to pSHIN-G except that the human H1 RNA promoter sequence is longer (215 nt rather than 91 nt) and works similarly to pSHIN-G. Six days after transfection, cells were immunostained and analyzed as described above.

## Detailed supplementary materials and methods

### Plasmid construction

pSHIN-G ( Small Hairpin Interfering Nucleotide expression) was constructed as below. The human H1 RNA promoter was PCR-amplified from p *Silencer*™ 3.1-H1 hygro (Ambion) with the following primers:

5′-GTGAATTCATATTTGCATGTCGCTATGTG-3′ and

5′-AAAAAGCTT **CCAAAAAATTGG**TT **CCATGGGAATGG**AGATCTGTGGTCCATACAGA-3′,

to introduce one *Bgl*II and two *Bst*XI (bold) sites. The amplified DNA fragment was cloned in pBluescriptII SK(-) (Stratagene) at *Eco*RI- *Hin*dIII sites and verified by DNA sequencing. Then the human H1 RNA promoter fragment was transferred to pME-EGFP4 similarly to vector construction of pG-SUPER. The pME-EGFP4 plasmid was created by self-ligation of pME-EGFP fragment digested with *Xba*I and *Spe*I, in order to eliminate these restriction enzyme sites at the 3′-UTR of EGFP.

The pSHIN-G[ *ccdB*] plasmid was created by insertion of a PCR-amplified *ccdB* gene to a *Bgl*II- *Hin*dIII gap of pSHIN-G. To erase a *Bst*XI site in the *ccdB* coding sequence, pENTR4 (Invitrogen) was mutated by QuikChange kit II (Stratagene) using two primers: 5′-CTGGCGCATGATGAC TACCGATATGGCCAG-3′ and 5′-ACTGGCCATATCGGT AGTCATCATGCGCCAG-3′.

The resulting plasmid was used as a template of PCR amplification of the *ccdB* DNA fragment using the following primers:

5′-AA **CCATTCCCATGG**GCTTACTAAAAGCCAGATA-3′ and

5′-AA **CCAAAAAATTGG**CTATATTCCCCAGAACATC-3′. The modified *ccdB* gene was inserted to a *Bst*XI gap of pSHIN-G.

The pREFLECT ( RNAi Evaluation by Fluorescence Level of Expressed Carrier of Target) plasmids were generated as described below. For pREFLECT-G, *Bst*XI sites were introduced by insertion of annealed oligonucleotides (5′-GGCCG **CCAAGCAAATGG**AA **CCATTTCGATGG**T-3′ and 5′-CTAGA **CCATCGAAATGG**TT **CCATTTGCTTGG**C-3′) to a *Not*I- *Xba*I gap of pME-EGFP. For pREFLECT-R(DsR), the EGFP part of pREFLECT-G was replaced with a mutated DsRed2 cDNA sequence using *Eco*RI and *Not*I sites. The mutation was introduced to pDsRed2-N1 (Clontech) by QuikChange kit II (Stratagene) with two primers

(5′-TGGTGGAGTTCAAGTC TATCTACATGGCCAAGA-3′ and

5′-TCTTGGCCATGT AGATAGACTTGAACTCCACCA-3′)

to eliminate a *Bst*XI site in the original coding sequence of DsRed2. The introduced mutation was silent mutation (TCC to TCT, encoding Ser). pREFLECT-R(mCh) was created by replacement of the EGFP part of pREFLECT-G with a mCherry cDNA sequence using *Eco*RI and *Not*I sites. The mCherry sequence was originated from pmCherry-N1, which was created by replacement of the EGFP sequence of pEGFP-N1 (Clontech) with a PCR-amplified mCherry cDNA fragment using *Age*I and *Bsr*GI sites. PCR was carried for pRSET-mCherry provided by Dr. R. Tsien (UCSF) using the following primers:

5′-CC ACCGGTCGCCACCATGGTGAGCAAGGGCGAGGAG-3′ and

5′-TTACT TGTACAGCTCGTCCAT-3′.

The pREFLECT vectors containing the *ccdB* sequence were created as follow. The *ccdB* fragment was PCR-amplified from pSHIN-G[ *ccdB*] using the primers

5′-AA **CCAAGCAAATGG**GCTTACTAAAAGCCAGA-3′ and

5′-AA **CCATCGAAATGG**CTATATTCCCCAGAAC-3′.

The amplified fragment was inserted to the 3′-UTRs of the fluorescent protein genes at *Bst*XI sites. The detailed configuration is show in Supplemental Figure 7B.

pREFLECT-R(DsR)-Vim-FL, which contained the full length of the coding sequence of human vimentin in the 3’-UTR of DsRed2, was constructed as below. The vimentin cDNA was amplified from pEGFP-vimentin (a gift of Dr. R. Goladman, Northwestern Univ.) using PCR primers,

5′- AAA **CCAAGCAAATGG**ATGGCCACCAGGTCCGTGTC-3′ and

5′- AAA **CCATCGAAATGG**TTCAAGGTCATCGTGATGCT-3′. The amplified fragment was digested with *Bst*XI and inserted to a *Bst*XI gap of pREFLECT-R(DsR)[ *ccdB*]

### The knockdown (KD) index for the reporter assay with two plasmids

Experimentally, we conducted four sets of co-transfection: (1) pSHIN-G-shRNA + pREFLECT-R-target, (2) pSHIN-G (no shRNA expression) + pREFLECT-R-target, (3) pSHIN-G-shRNA + pREFLECT-R-control, and (4) pSHIN-G + pREFLECT-R-control. Here “target” means insertion of the 19-nt sequence of siRNA target, and “control” means insertion of a 19-nt scrambled sequence. If the medians of the R/G ratios in the experiments (1)–(4) are represented as m _1_–m _4_, respectively, the correction efficient, δ, is defined as m _4_/m _3_. This value symbolically means (R _control_/G _control_)/(R _control_/G _test_)=G _test_/G _control_ which corresponds to the ratio of the EGFP expression level of pSHIN-G empty and pSHIN-G-shRNA. If the knockdown does not occur, theoretically the expected median of experiment (1) will be calculated as;

m _1_(expected)=m _2_/δ which symbolically means (R _test_/G _control_)/(G _test_/G _control_)=R _test_/G _test_. Experimentally we confirmed that the m _2_/δ value could match the actual m _1_ very well (96+/-15%, n=4) without RNAi. The KD index means m _1_(observed)/m _1_(expected), because m _1_(observed)/m _1_(expected)=m _1_/(m _2_/δ)=m _1_/m _2_*δ (the definition of KD). Therefore the KD index corresponds to the ratio, red fluorescence (observed; with RNAi)/red fluorescence (expected; without RNAi). Symbolically, the KD index (m _1_(observed)/m _1_(expected)) can be expressed as (R _test_/G _test_) _with RNAi_/(R _test_/G _test_) _without RNAi_=R _test with RNAi_/R _test without RNAi_, if assuming G _test_ is not changed by siRNA. As a conclusion, the KD index can be interpreted as the remaining level of red fluorescent protein after RNAi.

### Background estimation

We estimated background fluorescence two ways. In the first way, multiple regions where transfectants did not exist were manually determined and measured for the EGFP and DsRed2 fluorescence. This method was laborious, since it takes time to draw the regions manually for each sample. The second way used samples that were not transfected. The EGFP and DsRed2 fluorescence intensities were measured as for transfected samples. The averages were used for the background values.

### Thresholding of EGFP fluorescence

The threshold values were determined by inspection of data sets of two or three images. Since the transfection efficiency was constantly less than 50%, we could roughly estimate the average (m) and standard deviation (σ) of the fluorescence intensity for untransfected cells. The cut-off value was set to exceed m+3*σ.
