# Supplementary material for: An image-based, dual fluorescence reporter assay to evaluate the efficacy of shRNA for gene silencing at the single-cell level
Source: F1000Res. 2014 Feb 19;3:60. [Version 1] doi: 10.12688/f1000research.3-60.v1 (PMC3983939; doi:10.12688/f1000research.3-60.v1)
Supplement: Peer review history

# Peer review: Referee response for version 1

**Published:** 2014-04-07

**Authors:** Wen Lu

**Affiliations:** 1 Department of Cell and Molecular Biology, Northwestern University Feinberg School of Medicine, Chicago, IL, USA

This article introduces a new dual fluorescence assay for quantitatively measuring the efficiency of siRNA-mediated mRNA knockdown in cultured cells. The authors used two fluorescent reporters to independently represent the levels of target gene expression and shRNA expression and then applied computational analysis to calculate the ratiometric fluorescence value between the two reporters as an index of siRNA efficiency. This new approach presents a precise measurement of siRNA knockdown efficiency by comparing the target gene expression level to shRNA expression level directly in single cells, and serves as a reliable method to test different shRNA sequences for siRNA knockdown. Overall it clearly provides at least three major advantages over the traditional methods (western blot, etc.) for siRNA knockdown evaluation:

1. It measures the siRNA knockdown efficiency in individual cells so that it can bypass the potential problem of obtaining ambitious results from transfected cells that express the siRNA at various levels. Furthermore, they used automated image acquisition and analysis, which makes the approach feasible for high-throughput screening.     
   
2. It significantly shortens the procedure of estimating siRNA efficiency. This method targets an exogenous reporter gene from a transfected vector, minimizing the concern of pre-synthesized endogenous proteins. Because of this, it takes less time to see siRNA effect after transfection (~24 hours) compared to the traditional methods (>48 hours).  
   
3. The authors also introduced a negative bacteria selective marker, the *ccdB* gene, at the site of plasmid insertion, which may eliminate the colony selection step and significantly simplify the cloning procedure. 

Here are some points that I hope the authors could address:

1. How can this method estimate the siRNA efficiency if the target sequences are embedded in a complicated secondary structure? The authors used 19-bp target sequence at the 3’UTR of a red fluorescent protein, and used the red fluorescent signal as a measurement of siRNA knockdown. In most cases this technique is sufficient, as the authors documented in this article. But there are some exceptions as the authors pointed out, for example, the Lmna-T6 target sequence (Figure 3C and 3D). I wonder whether the authors can incorporate more 5’ and 3’ target sequences into the pREFLECT vector to better test the efficiency at different local mRNA secondary structures. 
   
2. The advantage of a three plasmid system is not quite convincing. I think the true advantage of the two plasmid system is that the molar ratio of GFP and shRNA in each individual cell is fixed, and thus the GFP signal truly indicates how much shRNA is expressed in the same cell. By adopting the three plasmid system, this advantage is lost. The authors claimed that the three plasmid system can expedite the workflow because the GFP and mCherry vectors are essentially identical for all experiments, and the previously constructed shRNA vectors can be used without modification. Obviously, it could save some cloning time in constructing shRNA vector, but it creates issues in estimating shRNA expression level at the same time. For this reason, I do not see any significant advantage for testing siRNA targeting sequences using the three plasmid system over the two plasmid system.

I have read this submission. I believe that I have an appropriate level of expertise to confirm that it is of an acceptable scientific standard.

# Peer review: Referee response for version 1

**Published:** 2014-03-06

**Authors:** Kenneth Yamada, Matthew Kutys

**Affiliations:** 1 Laboratory of Cell & Developmental Biology, National Institute of Dental and Craniofacial Research, National Institutes of Health, Bethesda, MD, USA; 2 Laboratory of Cell & Developmental Biology, National Institute of Dental and Craniofacial Research, National Institutes of Health, Bethesda, USA

This article introduces a new, fluorescence-based reporter assay for evaluating the knockdown efficacy of individual siRNA sequences. The authors effectively demonstrate the robustness of their assay in predicting effective siRNAs by direct comparison to endogenous protein knockdown, and they highlight the advantages this dual reporter assay provides over traditional methods, including significantly faster result times (24 hours versus 48+ hours) and evaluation of knockdown on the individual cell level. To expedite plasmid construction, the authors additionally modified their reporter plasmids with negative selection markers to bypass the need for DNA sequencing after addition of specific siRNA/target oligos. This very promising dual fluorescence reporter assay will be accessible to most standard cell and molecular biology laboratories, provides clear advantages over traditional methods of siRNA evaluation, and will provide avenues for high-throughput screening for effective siRNA sequences.

Minor points:

1. The rationale behind the three plasmid system is a bit confusing. While it appears to be designed to allow adaptability with previously constructed shRNA plasmids, it seems that the limitation of losing direct shRNA dosage correlation to GFP expression could sometimes be an offsetting disadvantage. The expression of shRNA could be significantly different than that of GFP, especially in hard-to-transfect cell lines, where it could potentially lead to false positives/negatives in the R/G ratios.
   
2. Please clarify how the final bin of the R/G ratios are plotted, since the last bar in Figs. 2C, 5C, and 5D are all higher than adjacent bars. Does this last bin represent the sum of this particular X value and all higher values? Are these R/G final bins due to the “RNAi-insensitive cell” population mentioned on page 6?
   
3. On page 7, the authors note that EGFP expression was reduced by insertion of the hairpin sequence for some target sequences. How severe was this reduction in the worst cases?
   
4. In Fig. 3f, the tubulin control western blot staining does not seem to match the 10-fold dilution of WT at 1.8 compared to “neg” at 18 – which of the western blot kits was used, since it might not be linear?
   
5. Very minor text issues should be corrected on page 8 (“ *since mCherry maturates faster than that of DsRed2*”, page 11 (“ *unless noting specifically*”), and page 13 (“ *Image analysis was as follow*”).

We have read this submission. We believe that we have an appropriate level of expertise to confirm that it is of an acceptable scientific standard.
